# Identification of Cathepsin B as a Therapeutic Target for Ferroptosis of Macrophage after Spinal Cord Injury

**DOI:** 10.14336/AD.2023.0509

**Published:** 2024-02-01

**Authors:** Jiaqi Xu, Yinghe Ding, Chaoran Shi, Feifei Yuan, Xiaolong Sheng, Yudong Liu, Yong Xie, Hongbin Lu, Chunyue Duan, Jianzhong Hu, Liyuan Jiang

**Affiliations:** ^1^Department of Spine Surgery and Orthopaedics, Xiangya Hospital, Central South University, Changsha, 410008, Hunan Province, China.; ^2^Key Laboratory of Organ Injury, Aging and Regenerative Medicine of Hunan Province, Changsha, 410008, Hunan Province, China.; ^3^National Clinical Research Center for Geriatric Disorders, Xiangya Hospital, Central South University, Changsha, 410008, Hunan Province, China.; ^4^Department of Sports Medicine, Xiangya Hospital, Central South University, Changsha, 410008, Hunan Province, China.

**Keywords:** Spinal cord injury (SCI), Combined bioinformatic analysis, Ferroptosis, Cathepsin B, Protease inhibitor, M2 macrophage polarization

## Abstract

Hemorrhage and immune cell infiltration are the main pathological features of spinal cord injury (SCI). Excessive iron deposition is caused by leaking hemosiderin which may over-activate ferroptosis pathways, resulting in lipid peroxidation and mitochondrial dysfunction in cells. Inhibiting ferroptosis after SCI has been shown to aid functional recovery. However, the essential genes involved in cellular ferroptosis following SCI are still unknown. Here we show that *Ctsb* is a statistical significance gene by collecting multiple transcriptomic profiles and identifying differentially expressed ferroptosis-related genes, which are abundantly expressed in myeloid cells after SCI and widely distributed at the epicenter of the injury. The expression score of ferroptosis, calculated by ferroptosis driver/suppressor genes, was high in macrophages. Furthermore, we discovered that inhibiting cathepsin B (CTSB), specifically with a small-molecule drug, CA-074-methyl ester (CA-074-me), reduced lipid peroxidation and mitochondrial dysfunction in macrophages. We also found that alternatively activated M2-polarized macrophages are more susceptible to hemin-induced ferroptosis. Consequently, CA-074-me could reduce ferroptosis, induce M2 macrophage polarization, and promote the neurological function recovery of mice after SCI. Our study comprehensively analyzed the ferroptosis after SCI from the perspective of multiple transcriptomes and provided a novel molecular target for SCI treatment.

## INTRODUCTION

Traumatic spinal cord injury (SCI) can lead to persistent neurological deficits below the damaged spinal cord segments, which cause devastating consequences for patients [1]. However, the current clinical treatments for SCI, which include surgical decompression, neurotrophic factors, and synthetic rehabilitation, have limited efficacy. The main reason for this is that the mechanism of SCI is unknown, and there are no specific drugs for clinical treatment [2]. Studying the pathological micro-environment of SCI can help us uncover the cellular and molecular mechanisms of SCI repair and discover new therapeutic targets.

Ferroptosis is a new type of cell death that was discovered in recent years [3]. Its characteristic pathological manifestations are morphological changes in the mitochondria, including shrinkage, an increase in membrane density, and the reduction of cristae. This cell death process is initiated by large amounts of iron and lipid peroxidation deposition in cells. In recent years, multiple studies have shown that ferroptosis plays a crucial role in cerebral hemorrhage, cancer, and many other diseases [4]. Following SCI, parenchymal hemorrhage and demyelination are significant pathological features. And the hemorrhage can cause a large accumulation of red blood cells at the lesion's epicenter. Therefore, dead red blood cells release hemosiderin, resulting in an excessive iron deposition. Furthermore, demyelination always results in a large accumulation of myelin debris, most of which are phospholipids. These two major pathological features suggest that ferroptosis is important in SCI repair [5]. However, evidence concerning excessive iron deposition and ferroptosis after SCI is limited. And the cellular and molecular mechanisms of ferroptosis are still unknown.

Macrophage infiltration in the injured microenvironment is another important pathological feature of SCI [6]. Studies demonstrated that these infiltrating macrophages could be activated to the M1 type and M2 type. M1 macrophage could secret pro-inflammatory factors, and M2 macrophage could secret anti-inflammatory factors to regulate SCI repair [6]. Moreover, the other function of macrophage is that it can clear and engulf the debris, such as hemosiderin, myelin debris, and dead neurons in the injured microenvironment [7]. Excess iron and lipid would be deposited in macrophages as a result of phagocytosis. However, there have been few studies on the effects of excess iron and lipid accumulation on macrophages during the SCI repair process.

To summarize, it is worth investigating whether excess iron and lipid accumulation can cause ferroptosis of macrophage and thus affect SCI repair. Several studies have shown that inhibiting ferroptosis has neuroprotective effects and promotes functional recovery following SCI [8-11]. Trehalose [12], liproxstatin-1 [13], and zinc [14] are also protective against ferroptosis after SCI by reducing oxidative stress. Nevertheless, a comprehensive analysis of the critical cells and molecules involved in ferroptosis after SCI needs to be explored.

The transcriptome data, particularly the single-cell sequencing profile, can be used to explore cellular and molecular mechanisms [15]. A transcriptome view of the pathobiology of SCI helps decode complex cellular interaction and identify feasible drug targets [16]. The most recent single-cell dataset of SCI also provided valuable insights into different molecular mechanisms [17]. These findings have not been used to investigate the critical genes in ferroptosis following SCI or to screen for treatment drugs.

The current study used synchrotron radiation micro X-ray fluorescence spectroscopy (SR-µXF) to investigate iron accumulation in the microenvironment of SCI. Then, we comprehensively reveal the molecular regulatory network of ferroptosis after SCI using transcriptome data mining. Furthermore, we would utilize small-molecule drug that target specific molecules and assess their therapeutic effect on SCI.

## MATERIALS AND METHODS

### Determining the iron content via synchrotron radiation-based micro X-ray fluorescence (SR-µXRF)

Normal and injured spinal cord tissues were collected at different time points after injury. The spinal cord was excised, frozen, and cut into slices (15 µm thick) using a cryomicrotome (Thermo Fisher Scientific, USA). The specimens were mounted on Ultralene foil and freeze-dried. The elements in the injured areas of the mouse spinal cord were detected using the synchrotron radiation-based micro-X-ray fluorescence (SR-µXRF) technique at BL15U1, Shanghai Synchrotron Radiation Facility (SSRF), China. The specimens were fixed to the platform at a 45° angle to the incident SR X-ray beam, and a silicon drift detector (SSD) (Vortex, USA) was placed at a 90° angle to the incident SR X-ray beam for recording the fluorescent counts of the metals. The energy of the exciting beam was set to 10 keV. The specimens were fixed on a sample platform controlled by a motorized X-Y mapping stage and were continually scanned in steps of 4 µm. At each point of the line scan, a fluorescent spectrum was recorded for 12 s with a resolution of 4 µm × 2 µm. The PyMCA program analyzed the SR X-ray spectra [18]. The intensity maps for the elements in the injured area of the spinal cord were analyzed using the Igor Pro software (WaveMetrics, USA).

### Bioinformatic analysis of transcriptomic profiles

The bulk RNA sequencing data of the injured mouse spinal cord (GSE5296, GSE47681, GSE45376, GSE46695, and GSE180767) and the information on the corresponding annotation were retrieved from the GEO database (www.ncbi.nlm.nih.gov/geo/). All profiles were derived from the tissue samples of the spinal cord injury contusion model. For GSE5296, data from the site of impact was collected. For GSE47681, data from the wild-type mice were collected. Data from the sham group was set as comparative control. The differentially expressed genes (DEGs) from profiles by array were analyzed separately using the R (3.6.3) package "limma". DEGs from profiles by high throughput sequencing were analyzed using the R package "DESeq2". For datasets from the same platform (GSE5296, GSE47681), their data from the same days post-injury (dpi) were combined after removing the batch effect using the R package "sva". The samples from different dpi were compared to those in the sham or control groups. The Benjamini and Hochberg method was used to adjust the p-value. Genes with adjusted p <0.05 and fold change ≥1 were included as DEGs for Gene Set Enrichment Analysis (GSEA) using the R package "clusterProfiler". The enriched terms with adjusted p < 0.05 and false discovery rate (FDR) < 0.25 were considered to be significant.

In total, 115 ferroptosis driver genes and 86 ferroptosis suppressor genes which were tested in mice were retrieved from the FerrDb database [19] (https://zhounan.org/ferrdb) for analysis (Supplementary Table 1). By taking the intersection of these genes with the DEGs of each dataset, the driver and suppressor genes that were differentially expressed at different time points post-injury were screened and summarized. Based on the general pathophysiological characteristics of spinal cord injury in mice [6, 20], < 3 dpi was defined as the acute phase, 7 dpi was defined as the subacute phase, and 28 dpi was defined as the chronic phase. All ferroptosis-related DEGs from different datasets were unioned according to the phase. Gene Ontology (GO) and Kyoto Encyclopedia of Genes and Genomes (KEGG) pathway enrichment of all ferroptosis driver/suppressor genes were performed using the R package "clusterProfiler". Enriched terms with adjusted p < 0.05 were considered to be significant.

The normalized RNA expression data of human peripheral white blood cells from 58 samples were retrieved from GSE151371 [21]. The expression of *Ctsb*, the ASIA Impairment Scale (AIS), and the Injury Severity Score (ISS) of all samples were extracted. Samples were divided into four groups (healthy controls, trauma controls, AIS grade A, and AIS grade BCD). The expressions of *Ctsb* in each group were compared. The correlation between ISS and the expression of *Ctsb* in SCI patients was calculated by the Spearman correlation coefficient.

### Bioinformatic analysis of the single-cell transcriptomic dataset

The single-cell RNA sequencing data of the contusive mouse spinal cord injury (GSE162610) and the information on the corresponding annotation were retrieved from the GEO database. In this data (GSE162610), 8-mm segments of spinal cord at the center of injury site (or the same position in unjury tissues) were dissected and processed for single-cell suspensions. Samples were subsequently prepared, indexed and constructed final libraries for Illumina sequencing as the authors described [17]. CellRanger (v2-4) software was used to transform the Illumina output into gene-barcode count matrices which was read for further anlysis in our study. Data processing and analysis were performed using the R package "Seurat" [22]. The genes expressed in less than 10 cells were excluded. The gene expression matrix was normalized and scaled. We selected the top 20 principal components by performing PCA based on 3,000 variable genes. The "harmony" algorithm was applied to correct the batch effect [23]. The FindNeighbors() and FindClusters() functions were used to cluster cells on a shared-nearest-neighbor graph. We generated UMAP plots to visualize annotated cell clusters. The markers of each cell type were calculated using the FindAllMarkers() function, which compares gene expression between clusters by performing the Wilcoxon rank-sum test. The single-nucleus RNA sequencing data of the human adult spinal cord from seven donors (GSE190442) were also obtained and underwent consistent analysis workflow.

To achieve the quantitative evaluation of the ferroptosis level at single-cell resolution, 115 ferroptosis drivers and 86 ferroptosis suppressors acquired from the FerrDb database were added to the AddModuleScore() function in R package "Seurat" to calculate the ferroptosis-driver scores and ferroptosis-suppressor scores, respectively. The former minus the latter equals the final ferroptosis score of each cell.

While analyzing the macrophage subsets, 12 clusters were calculated. Among them, 4 clusters (5, 7, 8, 9) with relatively low expression of *Ctsb* were defined as *Ctsb*-low macrophages. The rest of the clusters were defined as *Ctsb*-high macrophages. The Findmarkers() functions were used to identify marker genes for the *Ctsb*-high macrophages and *Ctsb*-low macrophages. The GO, KEGG pathway enrichment, and GSEA of the marker genes were performed as above.

### Ethics statement

All research protocols were approved by the Animal Ethics Committee of Central South University. Animal care and use during our experiment were conducted under the guidelines of the Administration Committee of Affairs Concerning Experimental Animals in Hunan Province, China.

### Animals

C57BL/6 mice (8-10 weeks old) kept in a specific pathogen-free (SPF) animal facility were used for this study. The transgenic Lyz2 ^tm1(cre)If^°/J mice (Stock No: 004781) were purchased from Jackson Laboratory. The animals were provided with ad libitum access to food and water and maintained in a 12:12 h light-dark cycle.

### Spinal cord contusive injury model

The surgical procedure of spinal cord contusive injury in mice was conducted as described in our previous study [24]. Briefly, animals were anesthetized by intraperitoneally injecting them with sublethal doses of 8 mg/kg ketamine and 10 mg/kg xylazine to minimize suffering during the surgery. Following anesthesia, the mice were fixed on a heating pad and underwent laminectomy. The spinal cord at T10 was exposed. A contusion injury (50 kdyne) was performed using an Infinite Horizon's impactor (Precision Systems Instrumentation) [25]. All mice were administered antibiotics (penicillin sodium; North China Pharmaceutical) for three days post-surgery. The bladders of the mice were manually pressed for seven days post-surgery.

### Drug administration in vivo

The animals were divided into three experimental groups: (1) The sham group, where laminectomy was conducted without spinal cord contusion. (2) The control group, where after laminectomy and spinal cord contusion, 100 µL of vehicle solvent (10% DMSO, 40% PEG300, 5% Tween-80, and 45% saline) was injected through the tail vein immediately after the operation and continued once a day for 14 consecutive days. (3) The treatment group, where after laminectomy and spinal cord contusion, 10 mg/kg CA-074-me (100 µL, MedChemExpress, HY-100350), dissolved in the vehicle, was injected through the tail vein immediately after the operation and continued once a day for 14 consecutive days.

### Cell culture and treatment

The mouse macrophage cell line, RAW 264.7, was purchased from the American type culture collection (Manassas, VA, USA) and cultured in 90% high glucose DMEM (Gibco) supplemented with 10% fetal bovine serum (FBS) (Gibco) in a humidified atmosphere with 5% CO_2_ at 37 °C. Upon treatment, the RAW264.7 cells were adjusted to a cell concentration of 5 × 10^4^/mL and plated in a six-well plate for 24 h. The cells were then incubated for 12 h in hemin. After incubation, DMSO or CA-074-me was added to the medium, and the cells were cultured for another 36 h.

For polarization of cells into M1/M2 states, RAW 264.7 macrophages were polarized by incubation in DMEM with 10% FBS, 50 U/ml penicillin-streptomycin containing interferon-γ (IFN-γ; 100 ng/ml; PeproTech; 315-05) for the M1 state or IL-4 (20 ng/ml; PeproTech; 214-14) for the M2 state for 48 h. After the polarization, cells were harvested, resuspended, and incubated in a six-well plate for 12 h in hemin. After incubation, DMSO or CA-074-me was added to the medium, and the cells were cultured for another 36 h.

### CCK-8 assay

The RAW264.7 cells were adjusted to a concentration of 5 × 10^4^/mL and plated 100 µl in a 96-well plate for 24 h. The cells were then incubated for 12 h in various concentrations of hemin (10, 20, 40, 80, and 160 µM). After incubation, DMSO or CA-074-me (10, 20, 40, 80, and 160 µM) was added to the medium, and the cells were cultured for 36 h. Cell viability was measured using the Cell Counting Kit-8 (Abcam, ab228554) method and a microplate reader (Thermo Fisher Scientific, USA) following the manufacturer's instructions.

### Immunohistochemistry, Prussian blue iron staining, and Oil Red O staining

The animals were anesthetized and transcardially perfused with 4% PFA. 8-mm segments of spinal cord at the center of injury site (or the same position in sham tissues) were collected and sectioned (30 µm thick). Immunohistochemistry of 4-Hydroxynonenal (4-HNE) and CTSB was conducted using the Super Plus™ High Sensitive and Rapid Immunohistochemical Kit (pH6.0) (Elabscience Biotechnology, E-IR-R221). The anti-4 HNE antibody and anti-CTSB antibody were used for labelling (Supplementary Table 2). Secondary antibody-only controls were employed to validate antibody specificity and distinguish genuine target staining from the background. Histopathological tissue staining was conducted using the Prussian Blue Iron staining kit (Solarbio Science & Technology, G1422) and Modified Oil Red O staining kit (Solarbio Science & Technology, G1261) following the manufacturer's instructions. The positively stained areas in the injured epicenter were quantitatively analyzed using the ImageJ software "IHC profiler."

### Western Blotting

Cells were lysed by the RIPA lysis buffer (CWbio, CW2333S) containing protease and phosphatase inhibitors (CWbio, CW2383, CW2200). After centrifugation at 12,000×g for 15 min, the supernatant was collected, and the protein concentration was determined using the BCA protein colorimetric assay kit (Elabscience Biotechnology, E-BC-K318-M). The supernatant was diluted with the SDS-PAGE protein loading buffer (5×; Beyotime Biotechnology, P0015) and heated at 100 °C for 10 min. Proteins were separated using 6%-10% SDS-PAGE gels depending on the molecular weight and transferred onto a polyvinylidene fluoride membrane (0.2 µm; Millipore). The membranes were blocked with 5% milk in 1×TBST (Solarbio) for one h and then incubated with primary antibodies, including anti-mtTFA, anti-ACSL4, anti-4-HNE, anti-iNOS, anti-Arg1, anti-IL-1 beta, and anti-β-actin (Supplementary Table 2). The blots were then rinsed three times with 1×TBST, each time for ten minutes, and incubated with species-appropriate secondary antibodies conjugated with peroxidase for 1 h. Rinse the blots again with 1×TBST for three times, each time for ten minutes. Finally, using the enhanced chemiluminescence reagent (ShareBio, SB-WB001), the immunoreactive bands were visualized with a ChemiDoc XRS Plus luminescent image analyzer (Bio-Rad, England). The blot images were quantitatively analyzed using the ImageJ software. Relative expression of all target proteins to β-actin was used for statistical comparison.

### Immunofluorescence assay

The animals were anesthetized and transcardially perfused first with 20ml saline and later with 30ml 4% paraformaldehyde. The spinal cords were collected and dehydrated with sucrose gradient for three days. Then the spinal cords were sectioned (30 µm thick) using a cryomicrotome (Thermo Fisher Scientific, USA). Cells on the cell climbing sheets were fixed with 4% poly-oxymethylene. Immunostaining analysis was performed following standard protocols. The sagittal spinal cord sections or cells were permeabilized with 0.5% Triton X-100 in PBS for 30 min and blocked with 5% bovine serum albumin (BSA) in PBS for 2 h. Following blocking, the sections or cells were incubated with primary antibodies, including anti-4-HNE, anti-Cathepsin B, anti-Lamp1, anti-CD11b, anti-CD68, anti-CD86, anti-CD206, anti-GFAP, anti-Fibronectin 1 antibodies, anti-F4/80, anti-IBA1, anti-iNOS, anti-Arg1, and anti-β-tubulin III overnight (Supplementary Table 2). After rinsing with 0.3% Tween-20 in PBS, they were incubated with the species-appropriate secondary antibodies conjugated with Alexa Fluor 488/ 594/ 647. The sections were then mounted on slides, covered with Vectashield DAPI Hardmount (Vector Laboratories), and examined under a fluorescence microscope or confocal microscope (Zeiss, Oberkochen, Germany). Secondary antibody-only controls were employed to validate antibody specificity and distinguish genuine target staining from the background. The images were quantitatively analyzed using the ImageJ software. The 3D reconstruction was performed using the Imaris 9.0 software.

### Transmission electron microscopy

The animals were anesthetized and transcardially perfused first with saline and then with 4% paraformaldehyde post-surgery. The spinal cord tissues in the injured center were immediately collected and stored in 2.5% glutaraldehyde for 4 h. The specimens were sliced (1 × 1 × 3 mm^3^) and double-fixed in 2.5% glutaraldehyde and shipped overnight at ambient temperature to the TEM laboratory at the Pathology Department of Xiangya Hospital, Changsha, Hunan, for further processing. During sample preparation, the samples were washed with Millonig's phosphate buffer (pH = 7.3), incubated for one h in 1% osmium tetroxide, and washed again. The samples were dehydrated at room temperature in an acetone gradient (50%, 70%, and 90% at 10-min intervals for each step, followed by 100% twice at 15-min intervals.). The specimens were then soaked in a mixture of acetone and resin (1:1) for 12 h and embedded with 100% resin overnight at 37 °C. During sample solidification, the specimens were polymerized with 100% resin overnight at 37 °C and then for 12 h at 60 °C. Ultrathin sections (90 nm) of spinal cord tissues were made with a UC-7 ultramicrotome (Leica) and a diamond knife. After double staining with 3% uranyl acetate and lead nitrate, the spinal cord sections were examined and photographed on a Hitachi HT-7700 electron microscope.

### MDA, LPO, and GSH assay

Approximately 0.5 cm of spinal cord tissue around the injured epicenter was harvested. After the tissue was cut, homogenized, and centrifuged, the supernatant was collected. The contents of MDA, LPO, and GSH were determined and calculated using the Malondialdehyde (MDA) colorimetric assay kit, the Lipid Peroxide (LPO) colorimetric assay kit, and the reduced glutathione (GSH) colorimetric assay kit (Elabscience Biotechnology, E-BC-K025-M, E-BC-K176-M, E-BC-K030-M), respectively, following the manufacturer's instructions. The sample was added to a 96 well plate and measured using a microplate reader (Thermo Fisher Scientific, USA).

### Measurement of Mitochondrial membrane potential (ΔΨm) and intracellular ROS levels

The cells seeded in the six-well plates were digested by trypsin and harvested at low temperatures. Cells were stained with JC-1 staining solution (Mitochondrial Membrane Potential Assay Kit containing JC-1, Elabscience Biotechnology, E-CK-A301) and then analyzed by flow cytometry according to the manufacturer's protocol. The data were quantitatively analyzed using FlowJo V10, as described in a previous study [26]. For ROS levels, Cells were stained with 2',7’-dichloro-fluorsecein-diacetate (DCFH-DA) from the Reactive Oxygen Species (ROS) assay kit (Beyotime Biotechnology, S0033S) and then analyzed by flow cytometry according to the manufacturer's protocol.

### Mitotracker Red staining

The RAW264.7 cells were incubated with Mitotracker Red CMXRos (Beyotime Biotechnology, C1035) for 30 min at 37 °C according to the manufacturer's protocol. The fluorescent images were acquired using a confocal microscope (Zeiss). The mitochondrial morphology was quantified using Image J.

### Behavioral and functional assessments

Two well-trained researchers, who were blinded to the experimental design, assessed the functional recovery of the hind limb motor function of mice before injury and post-SCI.

### BMS score and subscore evaluation

The left and right hind limb motor function of mice before the injury and on days 1, 3, 5, 7, 10, 14, 21, and 28 post-SCI was assessed using the open-field Basso Mouse Scale (BMS) score evaluation system as described in another study [27]. The BMS scores ranged from 0 (complete paralysis) to 9 (normal movement function of the hind limbs). To differentiate more precisely between the fine details of locomotion, the 11-point BMS subscore, which included frequency of plantar stepping, coordination, paw position, trunk stability, and tail position, was also assessed.

### Grid walking

The mice were trained to walk on a grid box (20 × 30 cm in size with 2 × 2 cm squares) for three consecutive days before surgery, as described in another study [28]. Before injury and on days 7, 14, 21, and 28 post-SCI, the mice were placed at the bottom of the grid box, which was adjusted at a 45° slope. The number of hind limb falling (errors) from the grid for the duration of 100 steps from the bottom to the top was counted. The trial was repeated three times for each mouse, and the average was recorded.

### Louisville swim scale swimming score

The mice were trained to swim in a water-filled tank (5 × 15 cm) from one side to the other for three consecutive days before surgery. On 28 dpi, the mice were placed in the tank for 30 s. The Louisville Swim Scale (LSS) swim score. which included hindlimb movement, hindlimb alternation, forelimb dependency, trunk stability, and body angle, was recorded as described in another study [29].

### Electrophysiology testing

Electromyography was performed to assess the motor-evoked potentials (MEPs) of the mice on 28 dpi, as described in our previous study [30]. The mice were anesthetized by intraperitoneally injecting them with a sublethal dose of 8 mg/kg ketamine and 10 mg/kg xylazine. The positive stimulating electrode was placed on the skull surface of the motor area of the cerebral cortex, and the negative stimulating electrode was placed on the skull near the orbital bones. The recording electrode was inserted into the left or right gastrocnemius muscle. The reference electrode was inserted into the distal tendon of the hind limb muscle. The ground electrode was placed under the skin of the back. A 3-mA single square wave (2 Hz) was provided for 0.2 ms to stimulate the mice. The amplitude of the hind limbs was recorded by calculating from the initiation point of the first response wave to its highest peak.

### Statistical analysis

The statistical analysis of the results was performed using GraphPad Prism (version 7.0, La Jolla, CA, USA). All data were reported as mean ±standard deviation (SD). Normality was determined using the Shapiro-Wilk test for n < 5 and the Kolmogorov-Smirnov test for n >= 5.

For two groups comparison, the homogeneity of variances was tested using the F-test. If the data followed a normal distribution and homogeneity of variance, an unpaired t-test was conducted for the statistical analysis. If the data did not follow a normal distribution, the Mann-Whitney test was used. If the data followed a normal distribution but did not meet the homogeneity of variances assumption, Welch's test was used.

For multiple groups comparison, the homogeneity of variances was tested using the Brown-Forsythe test. If the data followed a normal distribution and homogeneity of variance, an ordinary one-way ANOVA and Tukey's multiple comparisons test were performed for the statistical analysis. If the data did not follow a normal distribution or if the sample size was too small (n=3), the Kruskal-Wallis test and Dunn’s multiple comparisons test was used. If the data followed a normal distribution but did not meet the homogeneity of variances assumption, the Brown-Forsythe and Welch ANOVA tests were used.

All differences among and between groups were considered statistically significant at p < 0.05. In the figures, ns denotes p ≥ 0.05, * denotes p < 0.05, ** denotes p < 0.01, *** denotes p < 0.001, and **** denotes p < 0.0001. Supplementary Table 3 provides a detailed description of the statistical analysis procedures and the QQ plot.

**Figure 1.**
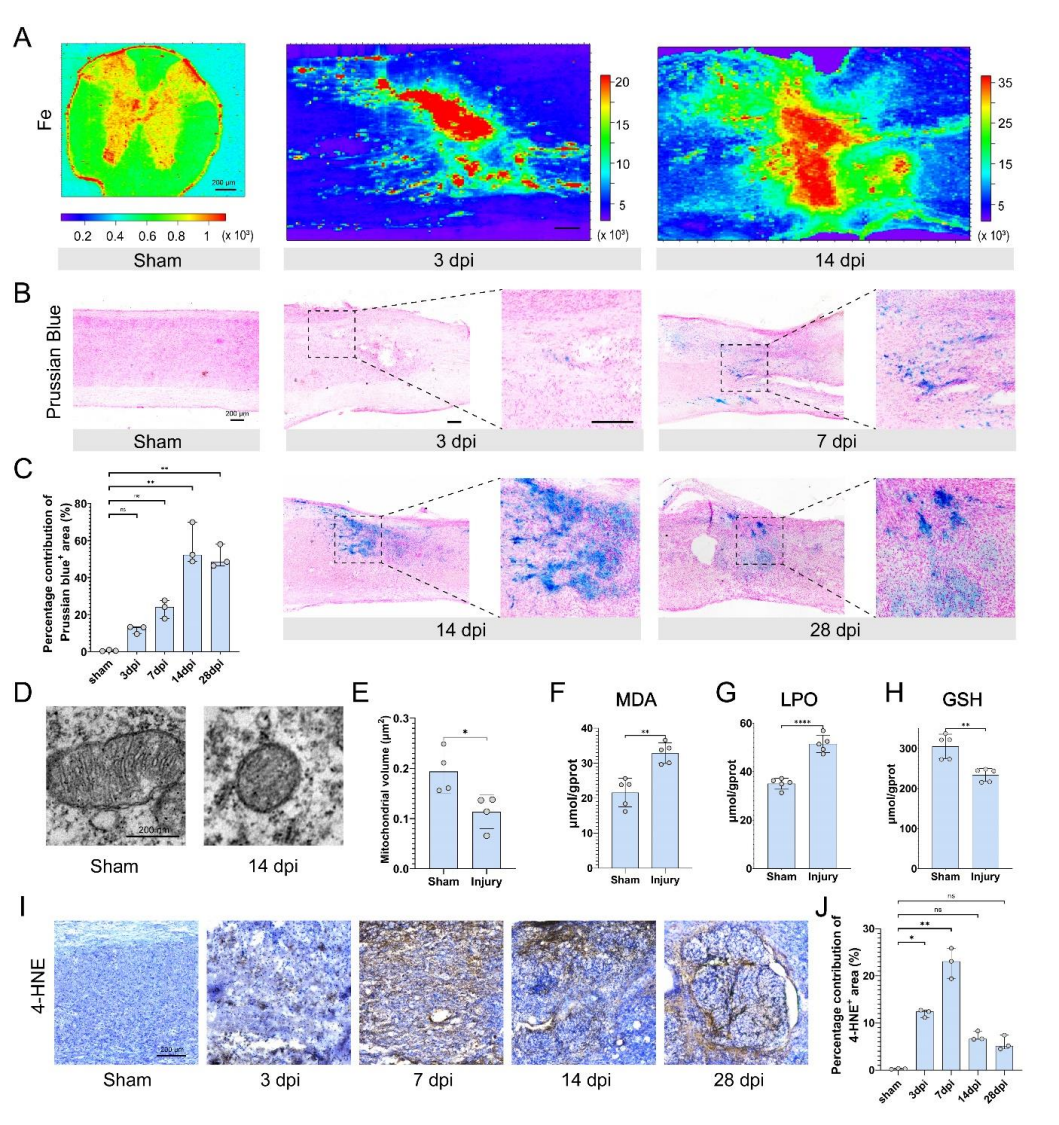
**Excessive accumulation of iron and lipid peroxide in the injured epicenter after SCI**. (**A**) Synchrotron radiation-based micro X-ray fluorescence (SR-µXRF) image of the iron content of the spinal cord in the sham group and at three dpi and 14 dpi. (Scale bar = 200 μm) (B) Prussian blue staining of the spinal cord in the sham group and at three dpi, seven dpi, 14 dpi, and 28 dpi. (Scale bar = 200 μm) (C) Quantification of the residual hemosiderin content in (B) (n=3, median with range, Kruskal-Wallis test, Dunn’s multiple comparisons, ns not significant, ** p < 0.01). (**D**) Representative Transmission Electron Microscope (TEM) images of mitochondria morphology in the sham group and at 14 dpi. (Scale bar = 200nm) (E) Quantification of the mitochondrial volume in (D) (n=4, mean ± SD, unpaired t test, * p < 0.05). (F-H) The content of Malondialdehyde (MDA), Lipid Peroxide (LPO), and reduced glutathione (GSH) in the spinal cord tissues in the sham group and at 14 dpi (n=5, mean ± SD, unpaired t test, ** p < 0.01, **** p < 0.0001). (**I**) Immunohistochemistry of 4-Hydroxynonenal (4-HNE) in the sham group, and at three dpi, 7 dpi, 14 dpi, and 28 dpi. (Scale bar = 200 μm) (J) Quantification of the 4-HNE^+^ area in (I) (n=3, median with range, Kruskal-Wallis test, Dunn’s multiple comparisons, ns not significant, * p < 0.05, ** p < 0.01).

**Figure 2.**
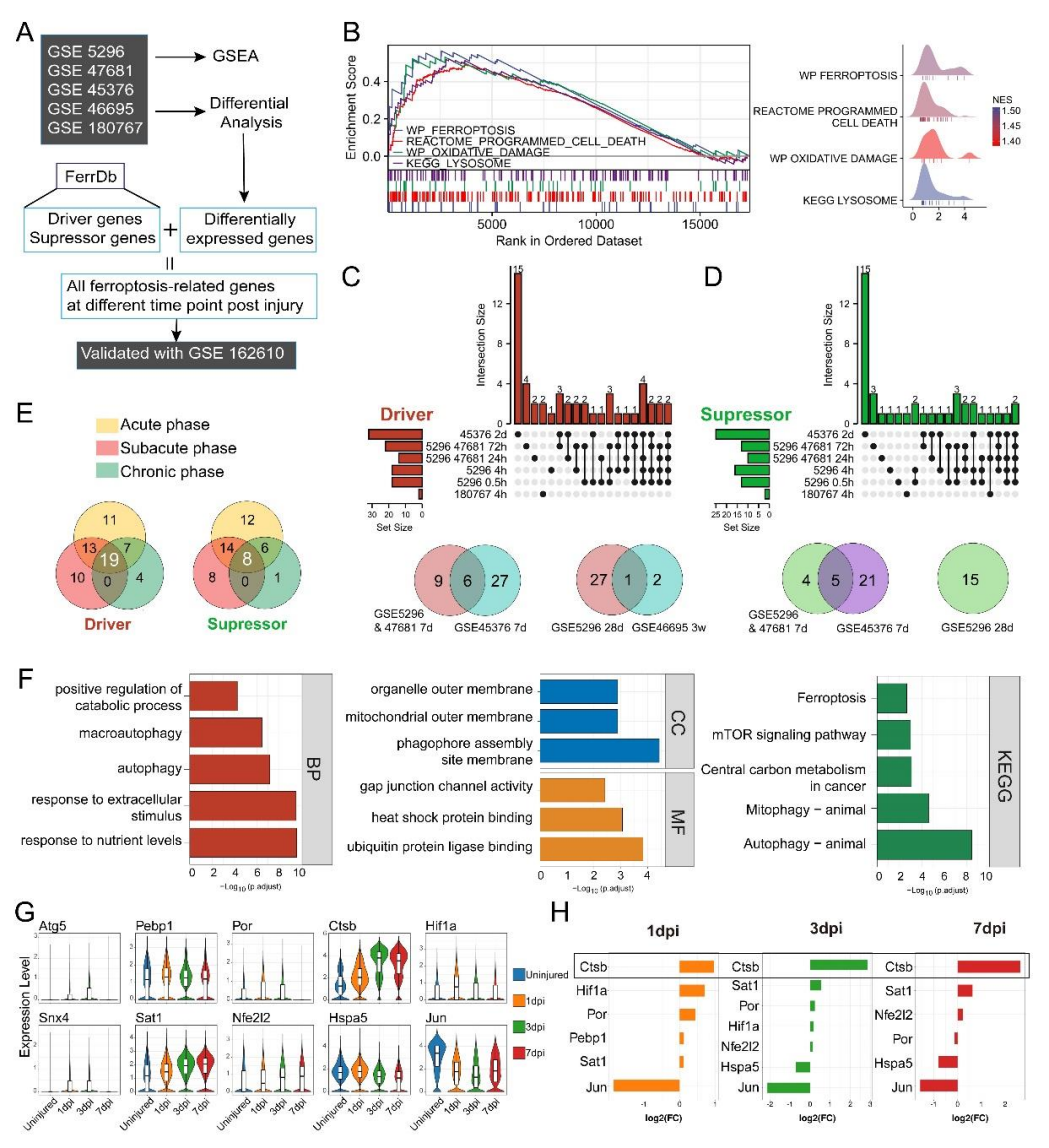
**Identification of differentially expressed ferroptosis-related genes from transcriptome profiles**. (**A**) Flowchart of combined *transcriptome* analysis. (**B**) GSEA visualization of pathways enriched in GSE5296 and GSE47681 at three dpi (injury versus sham). (C-D) Upset plot and Venn plot showing the union of ferroptosis driver/suppressor genes at the acute phase, subacute phase, and chronic phase from all datasets. (**E**) Venn plot showing the number of ferroptosis driver/suppressor genes in different phases. (**F**) Histogram showing the -log_10_ (p. adjust) of terms enriched by the GO and KEGG analysis of differentially-expressed ferroptosis driver/suppressor genes. (**G**) The expression level of 10 notable ferroptosis driver/suppressor genes in single-cell dataset GSE162610. (**H**) The log2 (Foldchange) value of differentially expressed ferroptosis driver/suppressor genes at one dpi, three dpi, and seven dpi in single-cell dataset GSE162610.

## RESULTS

### Excessive accumulation of iron and lipid peroxide in the injured epicenter after SCI

The concentration of iron, copper, and zinc in the spinal cord was measured using SRµXRF before and after injury at 3 and 14 dpi. The metal content was visualized using color gradient. Before injury, the spinal cord had low levels of metal elements, mostly concentrated in the grey matter (Fig. 1A, Supplementary Fig.1A-B). Iron increased at the injured epicenter at 3 dpi and was still abundant at 14 dpi but dispersed throughout the area (Fig. 1A). Copper and zinc content remained unchanged compared to pre-injury levels (Supplementary Fig. 1A-B). Prussian blue staining showed accumulation of hemosiderin in the epicenter (Fig. 1B), and residual hemosiderin content significantly increased after injury, particularly at 14 and 28 dpi (Fig. 1C). Oil red O staining showed significant lipid deposition after injury (Supplementary Fig. 1C-D). Transmission electron microscopy (TEM) images revealed that in the epicenter, mitochondria shrank, cristae disordered, and the matrix density between cristae increased (Fig. 1D). The volume of mitochondria decreased significantly at 14 dpi after injury (Fig. 1E). In addition to that, MDA and LPO levels in tissues also increased (Fig. 1F-G), accompanied by GSH depletion (Fig. 1H), and immunohistochemistry revealed the deposition of 4-HNE, a lipid peroxide, in the spinal cord. The findings demonstrated that 4-HNE accumulated and distributed in the epicenter (Fig. 1I). the 4-HNE^+^ area significantly increased after injury. At seven dpi, the 4-HNE^+^ area was the largest (Fig. 1J). Our results indicate the excessive accumulation of iron and lipid peroxide in the epicenter following SCI, indicating the occurrence of ferroptosis.

**Figure 3.**
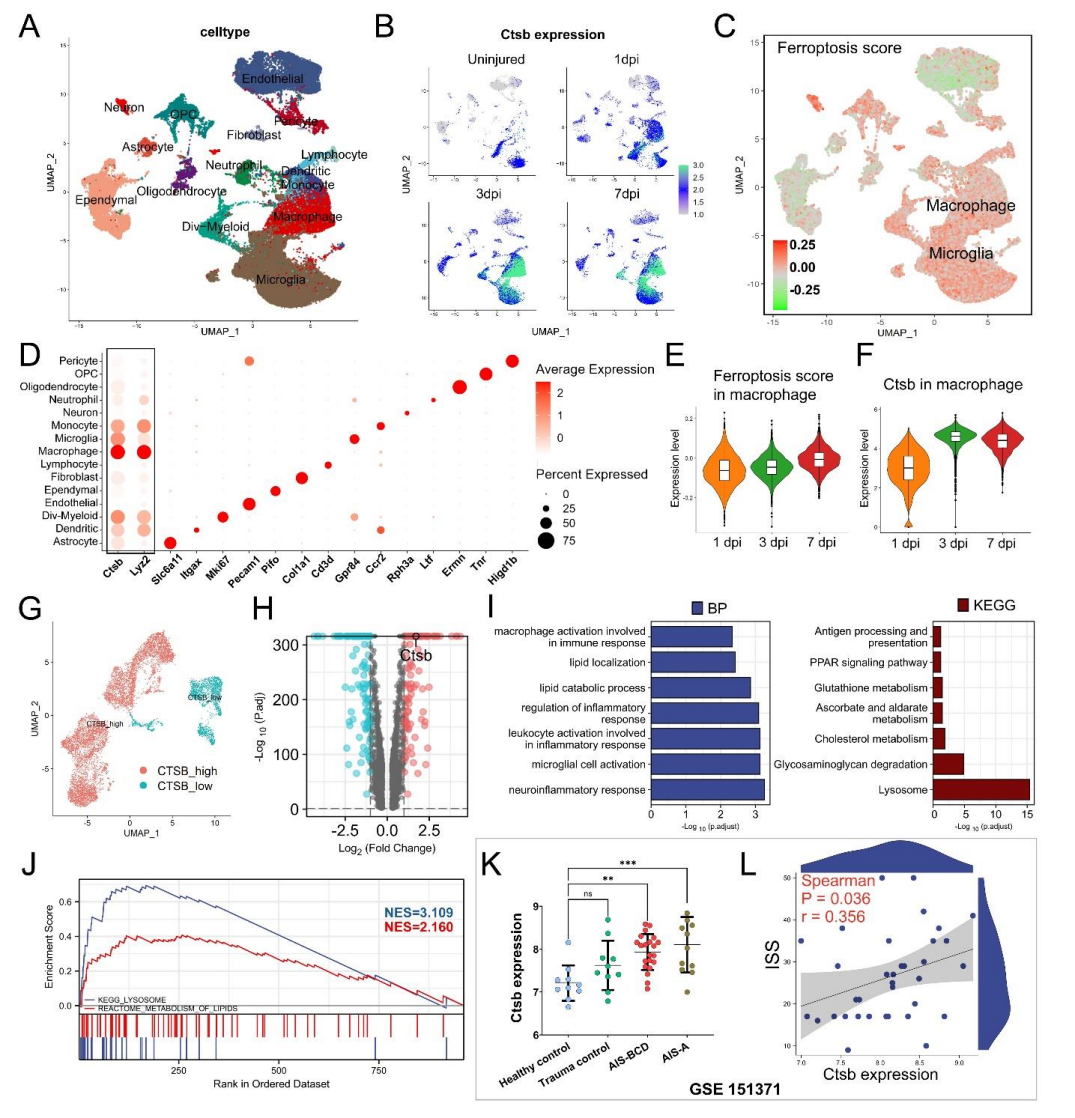
**Single-cell and peripheral white blood cells expression profiling of Ctsb in SCI**. (**A**) UMAP plot showing clusters and celltype annotations. (**B**) The expression level of *Ctsb* in different celltypes and time. (**C**) UMAP plot showing the calculated ferroptosis scores in different celltypes. (**D**) Dot plot showing the average expression of *Ctsb* and cell markers in annotated celltypes. (E-F) The ferroptosis scores and the expression level of *Ctsb* in macrophage cluster at one dpi, three dpi, and seven dpi. (**G**) UMAP plot showing two subtypes of macrophage based on *Ctsb* expression. (**H**) Volcano plot showing the distributions of DEGs between *Ctsb* -high macrophage and *Ctsb* -low macrophage. (**I**) Histogram showing the -log_10_(p.adjust) of terms enriched by the GO and KEGG analysis of DEGs between *Ctsb* -high macrophage and *Ctsb* -low macrophage. (**J**) GSEA visualization of pathways enriched in *Ctsb* -high macrophage. (**K**) The expression level of *Ctsb* in peripheral white blood cells from healthy control, trauma control, and SCI patients (AIS: ASIA Impairment Scale) (one-way ANOVA, Tukey's multiple comparisons, ns not significant, ** p < 0.01, *** p < 0.001. (**L**) The correlation scatter plot between Injury Severity Score (ISS) and *Ctsb* expression in peripheral white blood cells of SCI patients (Spearman correlation analysis, p=0.036, r=0.356).

### Identification of differentially expressed ferroptosis-related genes from transcriptome profiles

To investigate cellular ferroptosis in the injured microenvironment, we identified ferroptosis driver/ suppressor genes from differentially expressed genes (DEGs) after injury. A flowchart was utilized for the analysis (Fig. 2A). We used GSEA to demonstrate that ferroptosis-related pathways or the Reactome were enriched after SCI. At one dpi, ferroptosis, transferrin endocytosis and recycling, and biological oxidations were enhanced (Supplementary Fig. 2A). At three dpi, pathways, including ferroptosis, programmed cell death, oxidative damage, and lysosome, were involved (Fig. 2B). At seven dpi, programmed cell death and lysosome were enriched (Supplementary Fig. 2B). By intersecting all ferroptosis-related genes in DEGs at different dpi with driver/suppressor genes from FerrDb (Supplementary Fig. 2C-D), we grouped all ferroptosis driver and suppressor genes in a given phase together, dividing post-injury time points into three pathophysiological phases (Fig. 2C-D). Differentially expressed ferroptosis driver/suppressor genes at each time point were listed (Supplementary Table 4), and the number of genes at each phase was shown (Fig. 2E, Supplementary Table 5).

In total, we identified 19 ferroptosis driver genes and eight suppressor genes differentially expressed (Fig. 2E). GO enrichment analysis revealed that they participated in biological processes such as positive regulation of the catabolic process, macroautophagy, and autophagy, and were components of the phagophore assembly site membrane and mitochondrial outer membrane. The KEGG analysis showed their involvement in pathways, including ferroptosis, mTOR signaling, mitophagy, and autophagy (Fig. 2F). We confirmed their presence in the single-cell transcriptome profile GSE162610, listing ten genes with remarkable expression levels (Fig. 2G). Interestingly, *Ctsb* showed the most continuous and significant increase in expression after injury (Fig. 2H).

### Single-cell and peripheral white blood cells expression profiling of Ctsb in SCI

We further analyzed the dynamic expression of *Ctsb* at the single-cell resolution. The UMAP plot showed enhanced *Ctsb* expression levels in monocytes, dividing (div)-myeloid, macrophages, and microglia after SCI (Fig. 3A-B). Ferroptosis levels in all cell types were determined by calculating the expression score of ferroptosis-related genes in each cell, with macrophages and microglia showing higher levels of ferroptosis (Fig. 3C). The accuracy of cluster annotation was confirmed by the average expression level of *Ctsb* and canonical markers in different cell types. Macrophages exhibited the highest level of *Ctsb* expression, followed by div-myeloid cells, microglia, and monocyte, with *Ctsb* and *lyz2* showing similar expression patterns among cell types (Fig. 3D). Furthermore, both the ferroptosis score and *Ctsb* expression in macrophages increased considerably with injury progression (Fig. 3E-F).

To elucidate the features of macrophages with high *Ctsb* expression, we extracted macrophages and divided them into 12 clusters with optional resolution (Supplementary Fig. 3A-B). Based on *Ctsb* expression in these clusters, the whole-cell type was annotated as *Ctsb*-high or *Ctsb*-low macrophages (Fig. 3G). Their marker genes were calculated and ranked (Fig. 3H). GO enrichment analysis revealed that *Ctsb*-high macrophages were involved in biological processes such as immune response, lipid localization, lipid catabolic process, microglial cell activation, and neuroinflammatory response. KEGG analysis showed *Ctsb*-high macrophages were involved in pathways such as antigen processing and presentation, glutathione metabolism, and lysosome formation (Fig. 3I). GSEA indicated that pathways such as lysosome and lipid metabolism were enriched in *Ctsb*-high macrophages (Fig. 3J). These findings suggest that *Ctsb*, which is highly expressed in macrophages, is involved in ferroptosis, immune cell-mediated inflammatory processes, and abnormal lipid metabolism following SCI.

Additionally, the RNA sequencing of human peripheral white blood cells demonstrated a significant difference in *Ctsb* expression between healthy controls and SCI patients. The *Ctsb* expression was significantly higher in complete SCIs (AIS-A) and incomplete SCIs (AIS-BCD) than in healthy controls (Fig. 3K). Moreover, *Ctsb* expression was positively correlated with the ISS score in SCI patients (Spearman's r = 0.356, p = 0.036) (Fig. 3L), indicating that the level of *Ctsb* expression in human peripheral blood was associated with the severity of SCI injury. In another published single-nucleus RNA-sequencing dataset of the adult human spinal cord, 11 major clusters were mapped based on cell type annotation (Supplementary Fig. 3C). The feature plot and violin plot showed that *Ctsb* was exclusively enriched in microglia compared with other cell types, which mostly exhibited relatively low *Ctsb* expression (Supplementary Fig. 3D-E).

**Figure 4.**
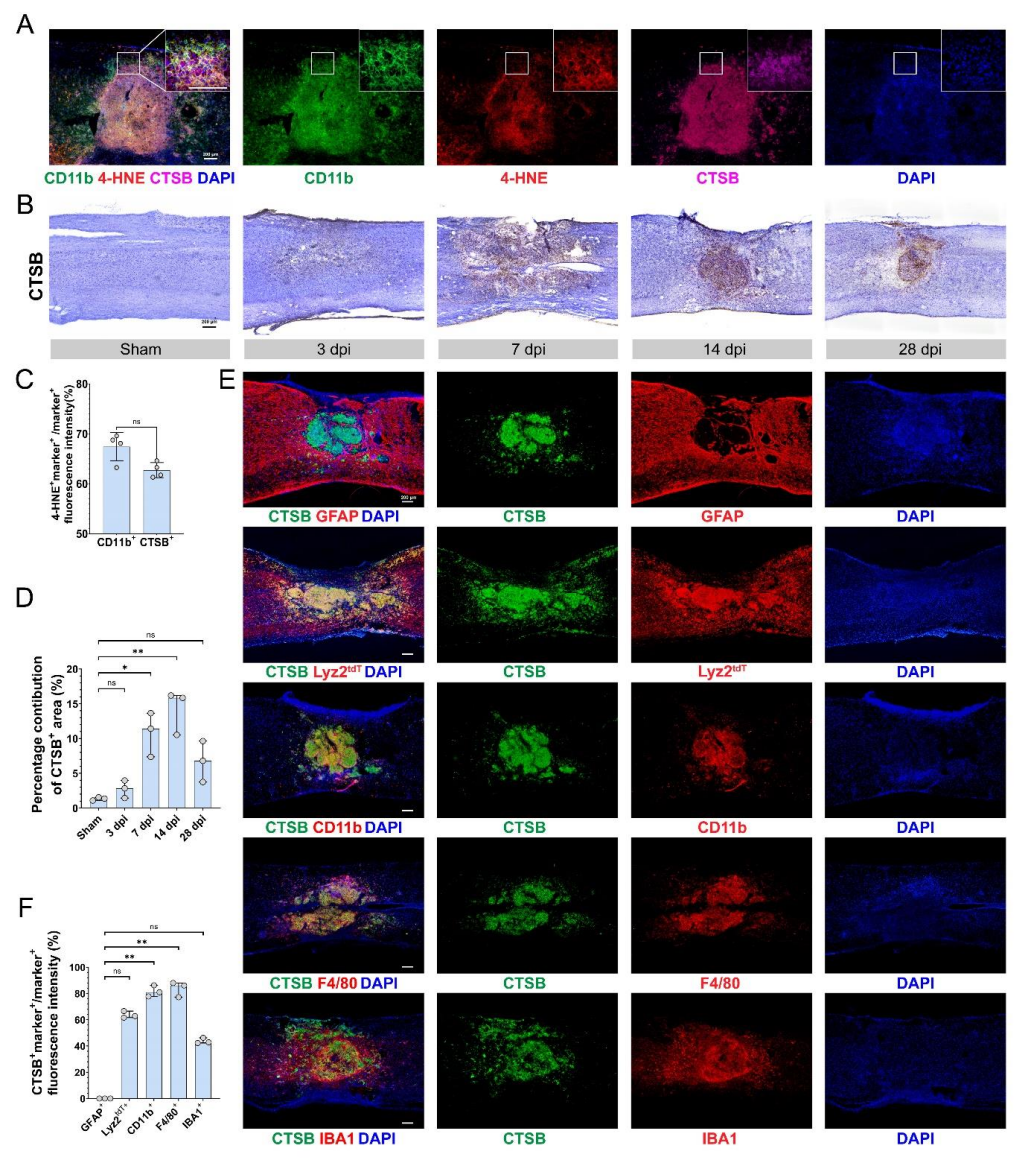
**CTSB is upregulated in myeloid cells after SCI**. (**A**) Representative immunofluorescence image and enlarged confocal image of CD11b (green), 4-HNE (red), CTSB (purple), and nucleus (blue) at 14 dpi (Scale bar = 200 μm). (**B**) Immunohistochemistry of CTSB in the sham group, and at three dpi, seven dpi, 14 dpi, and 28 dpi (Scale bar = 200 μm). (**C**) Quantification of the 4-HNE^+^ marker^+^ / marker^+^ area in (A) (n=4, mean ± SD, Mann-Whitnney test, ns not significant). (**D**) Quantification of the CTSB^+^ area in (B) (n=3, median with range, Kruskal-Wallis test, Dunn’s multiple comparisons, ns not significant, * p < 0.05, ** p < 0.01). (**E**) Representative immunofluorescence images showing the co-expression of GFAP (red), Lyz2^tdT^ (red), CD11b (red), F4/80 (red), and IBA1 (red)with CTSB (green) at 14 dpi. (**F**) Quantification of the CTSB^+^ marker^+^ / marker ^+^ area in (E) (n=3, median with range, Kruskal-Wallis test, Dunn’s multiple comparisons, ns not significant, ** p < 0.01) (Scale bar = 200 μm).

**Figure 5.**
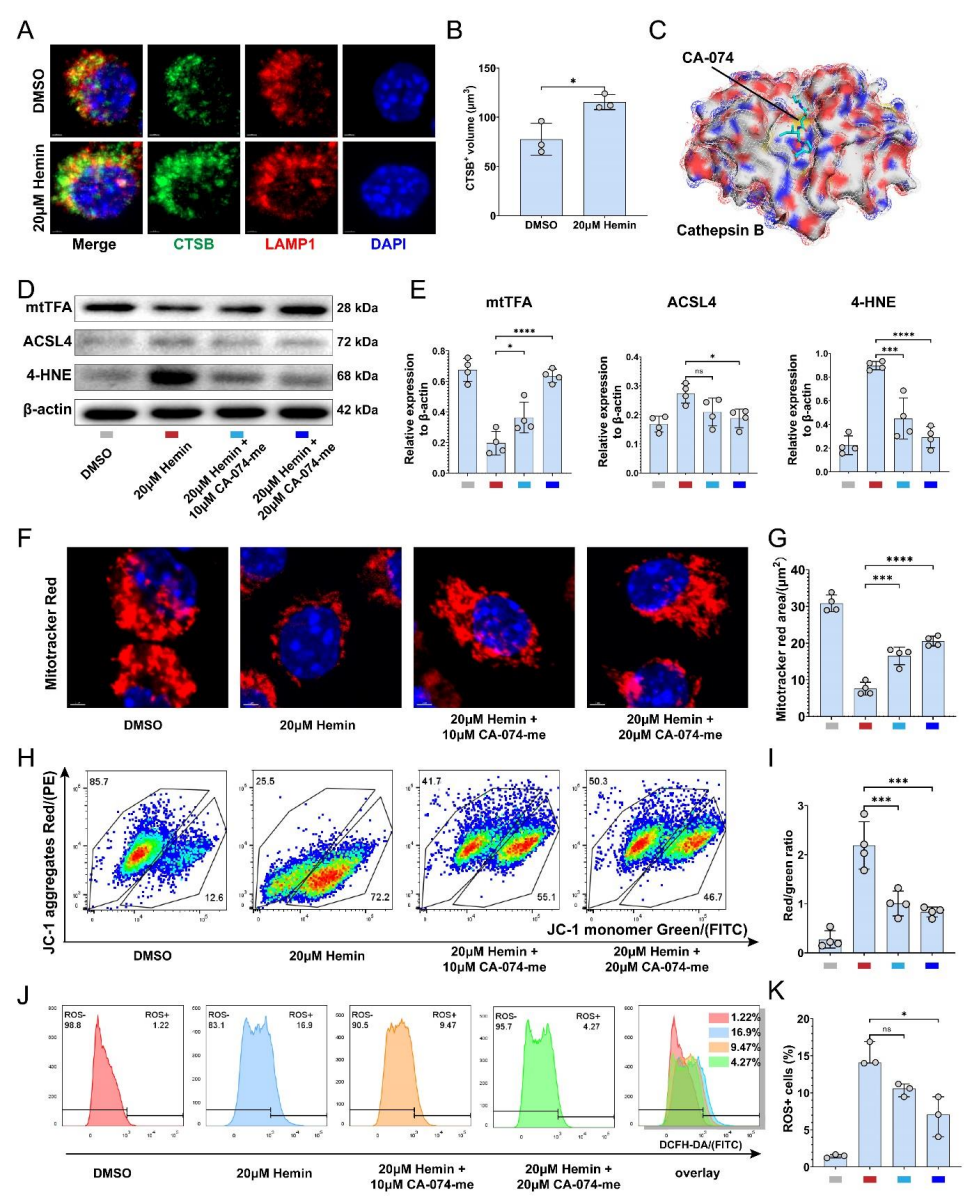
**CA-074-me alleviates the hemin-induced ferroptosis of macrophages in vitro**. (**A**) Representative immunofluorescence image of LAMP1 (red), CTSB (green), and nucleus (blue) after the treatment of DMSO or 20 µM hemin for 48h (Scale bar = 1 μm). (**B**) Quantification of the CTSB^+^ volume in (A) (n=3, mean ± SD, unpaired t test, * p < 0.05) (C) X-ray crystallography structure of the CTSB/CA-074 complex created with Pymol. (**D**) Western blotting analysis of the levels of mtTFA, ACSL4, 4-HNE, and β-actin in RAW 264.7 with different treatments. (**E**) Quantification of the relative expression of mtTFA, ACSL4, and 4-HNE to β-actin in (D) (n=4, mean ± SD, one-way ANOVA, Tukey's multiple comparisons, ns not significant, * p < 0.05, *** p < 0.001, **** p < 0.0001). (**F**) Representative confocal image of Mitotracker Red in RAW 264.7 with different treatments (Scale bar = 2 μm). (**G**) Quantification of the Mitotracker Red area per cell in RAW 264.7 (n=4, mean ± SD, one-way ANOVA, Tukey's multiple comparisons, ** p < 0.01, **** p < 0.0001). (**H**) Mitochondrial membrane potential indicated by flow cytometry of JC-1 staining. The gate in the upper left was set as normal cells. And the gate in the lower right corner was set to represent the cells with potential collapse. (**I**) Quantification of JC-1 mitochondrial membrane potential in (H) (n=4, mean ± SD, one-way ANOVA, Tukey's multiple comparisons, *** p < 0.001). (**J**) Intracellular ROS level indicated by flow cytometry of DCFH-DA staining. (**K**) Quantification of intracellular ROS level in (J) (n=3, median with range, Kruskal-Wallis test, Dunn’s multiple comparisons, ns not significant, * p < 0.05).

### CTSB is upregulated in myeloid cells after SCI

The qPCR analysis results demonstrated that the mRNA expression of *Ctsb* continuously increased after injury, peaked at three dpi, and gradually decreased after 14 dpi (Supplementary Fig. 4A). Immunofluorescence analysis revealed that a large number of CD11b^+^ myeloid cells in the epicenter expressed CTSB and 4-HNE. A significant proportion of CD11b^+^ and CTSB^+^ cells expressed 4-HNE (Fig. 4A). The 3D reconstruction of confocal images confirmed the presence of CTSB and 4-HNE on CD11b^+^ cells (Fig. 4A). CTSB was observed inside the cells, whereas 4-HNE was mostly found on the cell surface, indicating the occurrence of lipid peroxidation in CTSB^+^ macrophages. CTSB deposition in the injured area began at three dpi, was widely dispersed at seven dpi, and gradually concentrated towards the epicenter from 14 dpi to 28 dpi (Fig. 4B). There was no significant difference in 4-HNE expression between CTSB positive and CD11b positive cells (Fig. 4C). The content of CTSB in spinal cord tissue significantly increased after injury, with the highest levels observed at 14 dpi (Fig. 4D, Supplementary Fig. 4B). To further investigate CTSB expression in different cell types, we used the transgenic Lyz2 ^tm1(cre)If^°/J mouse and performed immunofluorescence assays to characterize CTSB expression in GFAP^+^ cells, Lyz2^+^ cells, CD11b^+^ cells, F4/80^+^ cells and IBA1^+^ cells (Fig. 4E). On average, 63.7% of Lyz2^+^ cells, 91.65% of CD11b^+^ cells, 83.78% of F4/80^+^ cells, and 43.77% of IBA1^+^ cells expressed CTSB. However, CTSB was not detected in GFAP-positive regions (Fig. 4F). These results confirmed that macrophages accumulate lipid peroxides after injury, and CTSB is highly expressed in macrophages, which is consistent with our transcriptome profiling findings.

### CA-074-me alleviates the hemin-induced ferroptosis of macrophages in vitro

To induce ferroptosis in RAW 264.7 macrophages, we used different concentrations of hemin, a commonly used ferroptosis inducer [31]. We found that a concentration of 20 µM was sufficient to cause stable cell death (Supplementary Fig. 5A), with hemin-treated cells displaying morphological irregularities and ruptured membranes (Supplementary Fig. 5B). Following treatment, there was an observed increase in the expression of CTSB around the Lamp1-labeled lysosomes (Fig. 5A-B). To investigate the role of CTSB in macrophage ferroptosis, a specific CTSB inhibitor, CA-074-me, was used. CA-074-me is a modified form of CA-074, with increased membrane permeability [32]. The structure of the CTSB/CA-074 complex (PDB ID: 1QDQ) was previously determined by X-ray crystallography by Yamamoto et al. [33], with Protein Contacts Atlas plots of ligands and residues depicting the immediate atomic contacts with amino acids (Supplementary Fig. 5C). We analyzed this structural data of the complex using PyMoL software for 3D visualization (Fig. 5C). At concentration below 40 µM, CA-074-me had no significant impact on cell viability (Supplementary Fig. 5D). Our findings revealed that CA-074-me was able to effectively reverse hemin-induced cell death at concentration higher than 10 µM, with a concentration of 20 µM having the best therapeutic effect on cell death (Supplementary Fig. 5E).

The cellular ferroptosis was assessed by measuring the protein expression of mitochondrial transcription factor A (mtTFA) and acyl-CoA synthetase long-chain family member 4 (ACSL4). MtTFA is a potential substrate of CTSB, and the leakage of CTSB from lysosomes can degrade mtTFA, leading to increased intracellular ROS [34]. On th other hand, ACSL4 promotes ferroptosis by catalyzing the synthesis of long-chain polyunsaturated CoAs [35]. In macrophages, hemin was observed to reduce mtTFA expression while elevating ACSL4 expression, along with increased production of 4-HNE. Notably, CA-074-me significantly reversed the changes in mtTFA, ACSL4, and 4-HNE levels caused by hemin (Fig. 5D-E). After hemin exposure, many cells exhibited a decrease in the area of mitotracker red and mitochondrial potential (Fig. 5F, H), while CA-074-me partially restored mitochondrial volume and potential in cells (Fig. 5G, I). The treatment of macrophages with CA-074-me also significantly decreased intracellular ROS levels (Fig. 5J-K). Our results indicate that CTSB inhibition can reduce ferroptosis *in vitro* by restoring mitochondrial function, reducing ROS production, and lipid peroxidation. 20 µM CA-074-me showed a stronger inhibitory effect on macrophage ferroptosis compared to 10 µM CA-074-me.

**Figure 6.**
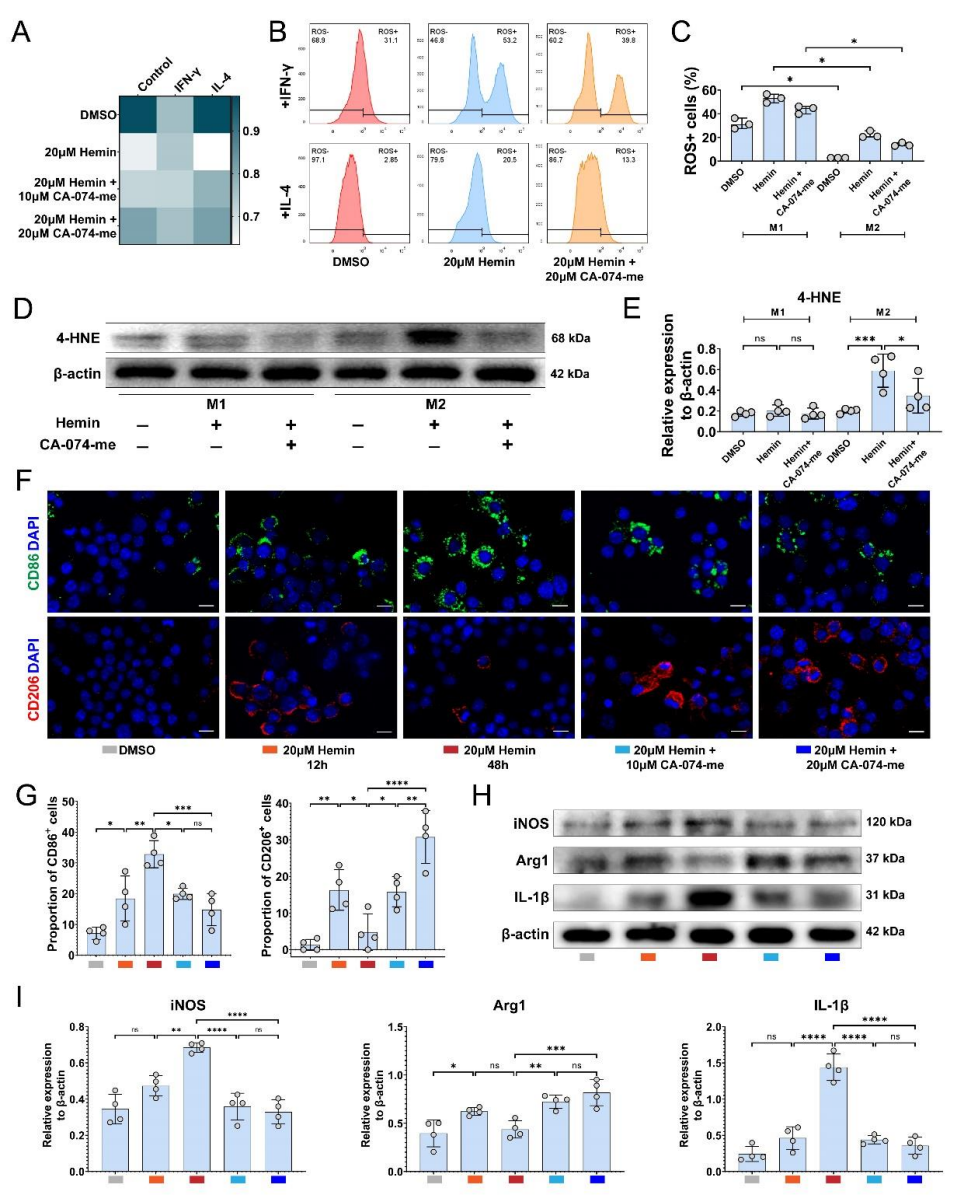
**Different sensitivity of M1 and M2 macrophages to ferroptosis is associated with the polarization state of macrophages**. (**A**) Cell viability of IFN-γ or IL-4 induced macrophage with different treatments (n=3, mean ± SD). (**B**) Intracellular ROS level of IFN-γ or IL-4 induced macrophage indicated by flow cytometry of DCFH-DA staining. (**C**) Quantification of intracellular ROS level in (B) (n=3, median with range, Kruskal-Wallis test, Dunn’s multiple comparisons, * p < 0.05). (**D**) Western blotting analysis of the levels of 4-HNE and β-actin in RAW 264.7. (**E**) Quantification of the relative expression of 4-HNE to β-actin in (D) (n=4, mean ± SD, one-way ANOVA, Tukey's multiple comparisons, ns not significant, * p < 0.05, *** p < 0.001). (**F**) Representative immunofluorescence image of CD86 (green) or CD206 (red), and nucleus (blue) in RAW 264.7 (Scale bar = 10 μm). (**G**) Quantification of the proportion of CD86/CD206 positive cells in (F) (n=4, mean ± SD, one-way ANOVA, Tukey's multiple comparisons, ns not significant, * p < 0.01, ** p < 0.01, *** p < 0.001, **** p < 0.0001). (**H**) Western blotting analysis of the levels of iNOS, Arg1, IL-1β, and β-actin in RAW 264.7. (**I**) Quantification of the relative expression of iNOS, Arg1, and IL-1β to β-actin in (H) (n=4, mean ± SD, one-way ANOVA, Tukey's multiple comparisons, ns not significant, * p < 0.05, ** p < 0.01, *** p < 0.001, **** p < 0.0001).

**Figure 7.**
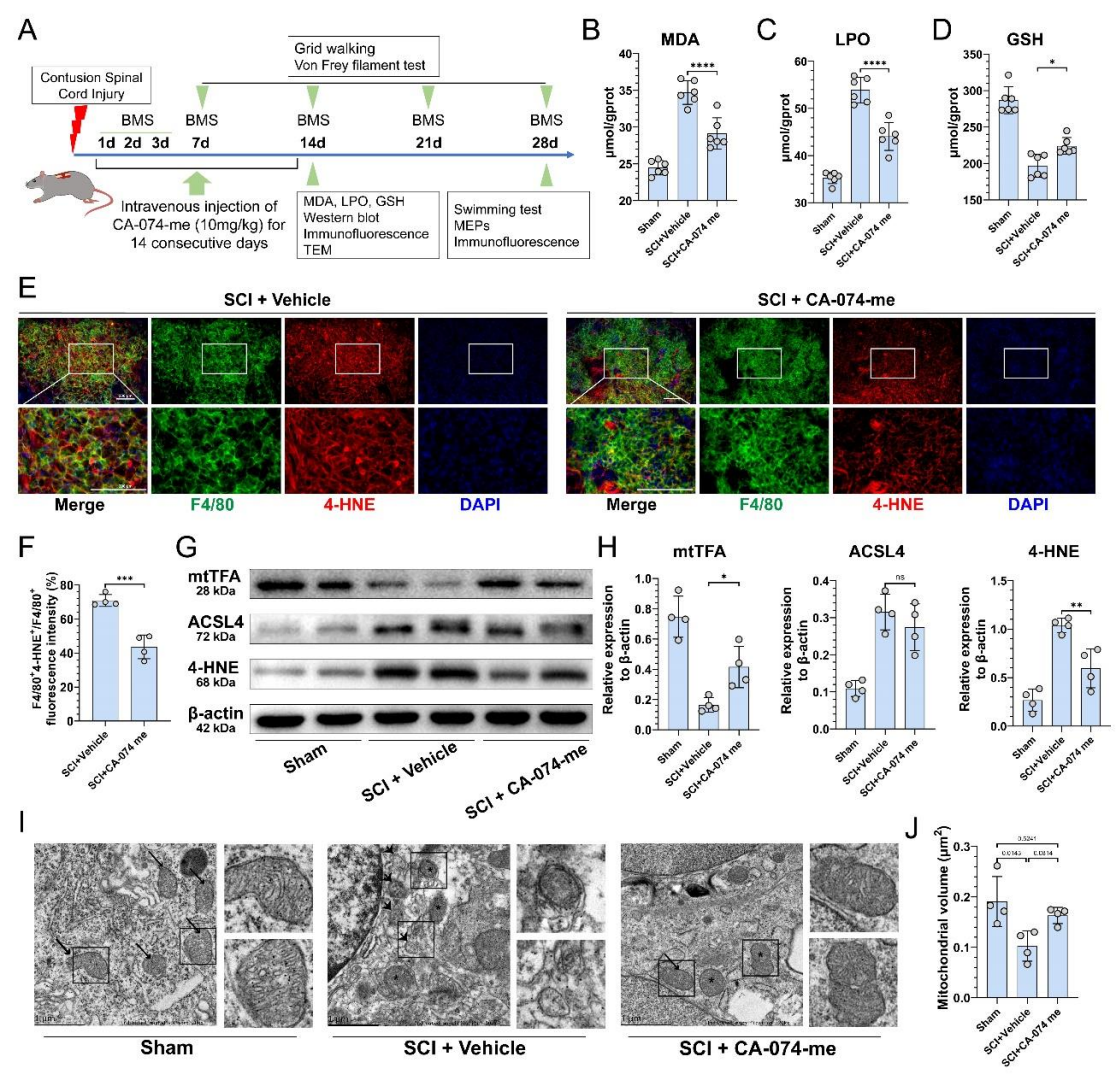
**CA-074-me reduces ferroptosis after SCI**. (**A**) Experimental design for the drug administration and evaluation process. (B-D) contents of MDA (B), LPO (C), and GSH (D) in the sham, control, and treatment groups (n=6, mean ± SD, one-way ANOVA, Tukey's multiple comparisons, * p < 0.05, **** p < 0.0001). (**E**) Representative immunofluorescence image of F4/80 (green), 4-HNE (red), and nucleus (blue) in the injured spinal cord at 14 dpi. (Scale bar = 200 μm) (F) Quantification of the F4/80^+^4-HNE^+^/F4/80^+^ fluorescence intensity in (E). (n=4, mean ± SD, unpaired t test, *** p<0.001) (G) Western blotting analysis of the levels of mtTFA, ACSL4, 4-HNE, and β-actin in the injured spinal cord tissues at 14 dpi. (**H**) Quantification of the relative expression of mtTFA, ACSL4, and 4-HNE to β-actin in (G) (n=4, mean ± SD, one-way ANOVA, Tukey's multiple comparisons, ns not significant, * p < 0.05, ** p < 0.01). (**I**) Representative Transmission Electron Microscope (TEM) images of mitochondria morphology in the sham, control, and treatment groups. Long arrows, normal mitochondria; Asterisk, shrunken mitochondria; Short arrows, vacuolized mitochondria. (Scale bar = 1 μm) (J) Quantification of the mitochondrial volume in (I) (n=4, mean ± SD, one-way ANOVA, Tukey's multiple comparisons, p value indicated in the figure).

### Different sensitivity of M1 and M2 macrophages to ferroptosis is associated with the polarization state of macrophages

In order to investigate the role of ferroptosis in macrophages following SCI, we examined the expression of *Cd86*^+^ M1-polarized macrophages and *Mrc1*^+^ (*Cd206*^+^) M2-polarized macrophages in GSE162610 (Supplementary Fig. 6A). The proportion of *Cd86*^+^ macrophages increased steadily from seven dpi, while the proportion of *Mrc1*^+^ macrophages gradually decreased (Supplementary Fig. 6B). Remarkably, the expression level of *Ctsb* in *Cd86*^+^ *Mrc1*^-^ macrophages and *Cd86*^-^ *Mrc1*^+^ macrophages showed no significant difference (Supplementary Fig. 6C), suggesting that *Ctsb* upregulation is consistent in both M1 or M2 macrophages. Given the higher resistance of M1 macrophages to ferroptosis [36], we investigated whether M2 macrophages were more susceptible to hemin-induced cell death. We first induced macrophages into M1 and M2 polarized macrophages using IFN-γ and IL-4, respectively (Supplementary Fig. 7A-C). We found that 20 µM hemin had no significant effect on the cell viability of M1 macrophages, whereas it induced cell death in M2 macrophages. Treatment with CA-074-me prevented M2 macrophage cell death (Fig. 6A). Following treatment, we measured the intracellular ROS level and 4-HNE content of M1 and M2 macrophages. Interestingly, hemin increased the ROS level in both M1 and M2 macrophages, with M1 macrophages having a higher level of ROS. Treatment with CA-074-me significantly reduced their ROS production (Fig. 6B-C). However, only hemin-induced M2 macrophages showed increased expression of 4-HNE, suggesting that M2 macrophages are more susceptible to lipid peroxidation and ferroptosis (Fig. 6D-E).

**Figure 8.**
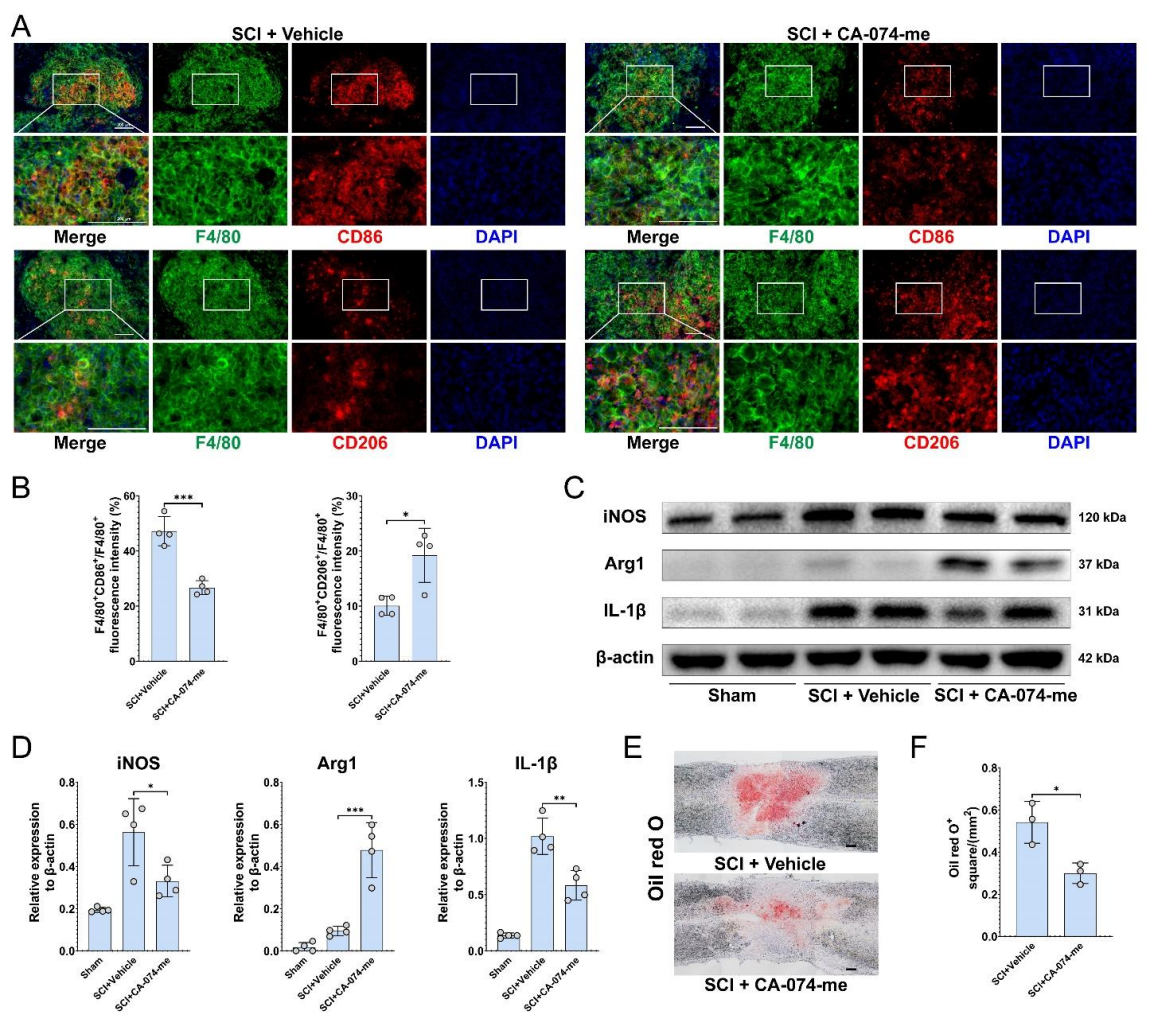
**CA-074-me promotes M2 polarization of macrophage after SCI**. (**A**) Representative immunofluorescence image of F4/80 (green), CD86 or CD206 (red), and nucleus (blue) in the injured spinal cord at 14 dpi. (Scale bar = 200 μm) (B) Quantification of the F4/80^+^CD86^+^ /F4/80^+^(unpaired t test) and F4/80^+^CD206 ^+^/F4/80^+^(Mann-Whitnney test) ^fluorescence^ intensity in (A) (n=4, mean ± SD, * p < 0.05, *** p <0.001). (**C**) Western blotting analysis of the levels of iNOS, Arg1, IL-1β and β-actin in the injured spinal cord at 14 dpi. (**D**) Quantification of the relative expression of iNOS, Arg1, and IL-1β to β-actin in (C) (n=4, mean ± SD, one-way ANOVA, Tukey's multiple comparisons, * p < 0.05, ** p < 0.01, *** p < 0.001). (**E**) Oil red O staining of the lipid content in the control and treatment groups at 14 dpi. (Scale bar = 200 μm) (F) Quantification of the Oil red O square in (E) (n=3, mean ± SD, unpaired t test, * p < 0.05).

Following treatment with 20 µM hemin for 12 h, both CD86^+^ and CD206^+^ macrophages were detected. However, after 48 h of exposure to hemin, the proportion of CD86^+^ cells and protein levels of iNOS and IL-1β continued to increase, while the proportion of CD206^+^ cells and protein levels of Arg1 decreased. Treatment with CA-074-me in combination with hemin significantly decreased the proportion of CD86^+^ cells and increased the proportion of CD206^+^ cells (Fig. 6F-G). Additionally, protein levels of iNOS and IL-1β were reduced, and the level of Arg1 was elevated after administering CA-074-me. (Fig. 6H-I). These findings suggest that M2 macrophages were more susceptible to ferroptosis than M1 macrophages, and CA-074-me can reduce the ferroptosis of M2 macrophages *in vitro*, leading to an increased proportion of M2 macrophages.

### CA-074-me reduces ferroptosis after SCI

Prior studies have indicated that chemical protease inhibitors administered exogenously may improve the recovery process following SCI [37]. In light of this, we explored the therapeutic efficacy of CA-074-me *in vivo*. The treatment and evaluation procedures are illustrated in Fig. 7A. MDA and LPO levels were significantly reduced after administration of CA-074-me (Fig. 7B-C), and the treatment group displayed slightly higher levels of GSH, which had decreased following SCI (Fig. 7D). Immunofluorescence assays showed that the expression of 4-HNE in F4/80-positive macrophages was considerably reduced following CA-074-me treatment (Fig. 7E-F). The protein level of mtTFA in the vehicle group was significantly lower, while CA-074-me significantly slowed its degradation. ACSL4 protein levels were slightly lower in the treatment group, and consistent with the changes in MDA and LPO, 4-HNE production was significantly reduced (Fig. 7G-H). Finally, mitochondrial TEM images revealed that in the sham group, mitochondrial morphology was normal with clear cristae (Fig. 7I). Within the injury epicenter, certain mitochondria exhibited shrinkage, with a disordered structure of the cristae, and an increased density of the matrix between cristae. In contrast, other mitochondria showed vacuolization, with a short and broken ridge structure, a collapsed membrane, and a lower electron density of the matrix, resulting in transparent vacuoles (Fig. 7I). In the treatment group, the structure of the cristae of shrunken mitochondria partially recovered, with a lower incidence of vacuolized mitochondria (Fig. 7I). Additionally, the mean mitochondrial volume in the treatment group increased (Fig. 7J). In light of these findings, it can be concluded that CA-074-me has potential therapeutic benefits, as it effectively reduces lipid peroxidation and mitochondrial degeneration in the injured spinal cord.

### CA-074-me promotes M2 polarization of macrophage after SCI

The state of macrophage polarization after SCI plays a significant role in the local microenvironment [38], with M2-polarized macrophages aiding the recovery of SCI [39]. We investigated the polarization states of activated macrophages following SCI and found that a large number of F4/80^+^ cells gathered in the epicenter of injury, with most of the cells being CD86 positive and only a few being CD206 positive, consistent with the single-cell expression profile (Supplementary Fig. 6B) (Fig. 8A). However, in the treatment group, the proportion of CD86^+^ macrophages was reduced, while the proportion of CD206^+^ macrophages significantly increased (Fig. 8A-B). In response to macrophage polarization, protein levels of iNOS and Arg1 also changed, with the protein level of IL-1β, a pro-inflammatory cytokine, being reduced in the treatment group (Fig. 8C-D). Macrophages act as scavengers of myelin debris [24], and M2-polarized macrophages usually exhibit high phagocytic activity [40]. Oil Red O staining also revealed that the lipid content in the injured area after SCI significantly decreased in the treatment group (Fig. 8E-F). These findings suggest that CA-074-me promotes M2 polarization of macrophages after SCI. Double staining of IBA1 and iNOS/Arg1 further confirmed a significant reduction in the iNOS expression of microglia and an increase in the Arg1 expression of microglia in the treatment group (Supplementary Fig. 8A-B).

### CA-074-me promotes functional recovery of mice after SCI

**Figure 9.**
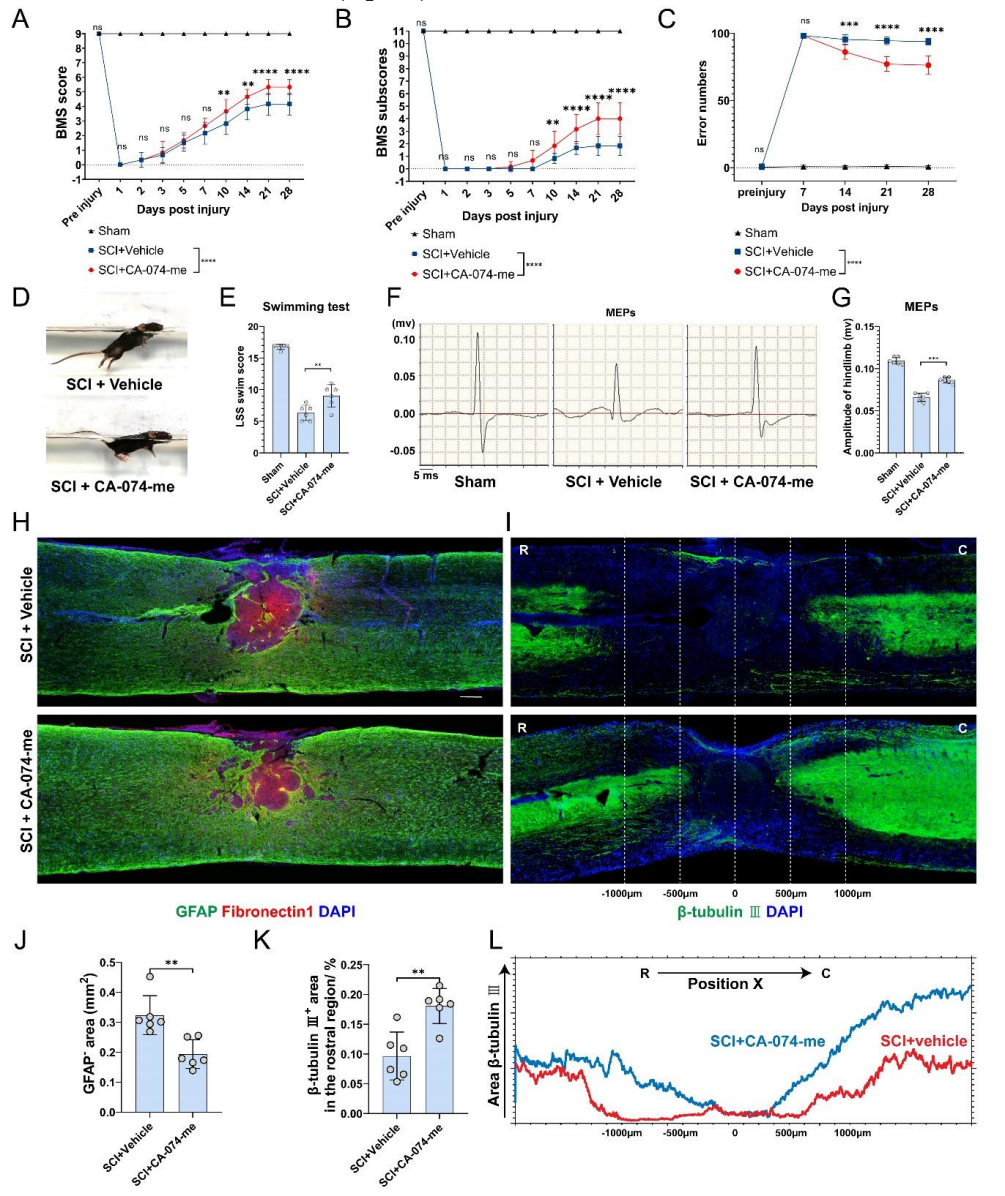
**CA-074-me promotes functional recovery of mice after SCI**. (A-C) Basso Mouse Scale (BMS) scores (A), BMS subscores (B), and error numbers of grid walking (C) over time post-injury in the sham, control, and treatment groups (n=6, mean ± SD, two-way ANOVA, Tukey's multiple comparisons, ns not significant, ** p < 0.01, *** p < 0.001, **** p < 0.0001). (**D**) Swim test at 28 dpi in the control and treatment groups. (**E**) Quantification of the swim test in (D) using the Louisville Swim Scale (LSS) swim score (n=6, mean ± SD, one-way ANOVA, Tukey's multiple comparisons, ** p < 0.01). (**F**) Representative images of motor-evoked potentials (MEPs) in the sham, control, and treatment groups at 28 dpi. (**G**) Quantification of the amplitude of hindlimb in (F) (n=6, mean ± SD, one-way ANOVA, Tukey's multiple comparisons, *** p < 0.001). (**H**) Representative fluorescence images of GFAP (green), Fibronectin 1(red), and nucleus (blue) in the sham, control, and treatment groups at 28 dpi. (Scale bar = 200 μm) (I) Representative fluorescence images of β-tubulin Ⅲ (green) and nucleus (blue) in the sham, control, and treatment groups at 28 dpi. (Scale bar = 200 μm, R: rostral, C: caudal) (J) Quantification of the GFAP negative scar area in (H) (n=6, mean ± SD, Mann-Whitney test, ** p < 0.01). (**K**) Quantification of the β-tubulin Ⅲ-positive neuronal fiber area in the rostral region in (J) (n=6, mean ± SD, unpaired t test, ** p < 0.01). (**L**) Curves showing the continuous distribution of β-tubulin Ⅲ-positive neuronal fiber area in (I).

To examine whether decreased ferroptosis could enhance functional recovery, several behavioral assessments were performed over four weeks. The BMS scoring system and grid walking test were used to assess hind limb motor function. The CA-074-me-treated group showed higher BMS scores (from 10 dpi), higher BMS subscores (from 10 dpi) and lower error numbers (from 14 dpi) compared to the control group (Fig. 9A-C). Treatment with CA-074-me administration resulted in a less inclined body angle, less pendent tail, and a more stable trunk (Fig. 9D-E), and increased amplitude of MEPs, as determined by the swimming test and electrophysiological examinations (Fig. 9F-G). Immunofluorescence staining of GFAP and fibronectin 1 showed that animals CA-074-me-treated animals had a smaller GFAP^-^ scar area at 28 dpi (Fig. 9H, J). Furthermore, the β-tubulin III-positive neuronal fibers in the rostral region of the treatment groups were significantly larger than those in the control groups at 28 dpi (Fig. 9I, K), indicating a reduction in axon retraction. Representative full-field views of consecutive β-tubulin III-positive neuronal fiber distribution (Fig. 9L) were further quantified. These results indicated that the inhibition of CTSB by CA-074-me effectively promoted tissue repair and neurological function recovery after SCI.

## DISCUSSION

A novel method, SR-μXRF, was used in this study to visualize a high-resolution atlas of iron accumulation in the injured area of SCI [41]. Subsequently, we first revealed that CTSB plays an important role in regulating ferroptosis in macrophages after SCI. Moreover, our study also demonstrated a link between the ferroptosis of macrophages and the neurological function recovery of SCI by identifying M2-type macrophages are ferroptosis-sensitive cells. Hence, ferroptosis can rapidly degrade the neuroprotective function of M2-type macrophages and hinder the recovery of SCI [42]. Finally, we confirm that CA-074, a specific inhibitor of CTSB, can inhibit cellular ferroptosis in a mouse SCI model and increase the proportion of M2-type macrophages in the injured area, promoting neural functional recovery in SCI mice.

Studying the injured microenvironment of SCI can help us uncover the pathological mechanism of SCI. In the present study, we confirmed the excessive iron accumulated in the pathological microenvironment of SCI. Blood components can infiltrate into the spinal cord tissue as a result of parenchymal hemorrhage. Leaking red blood cells, among other components in the blood, can release large amounts of hemin, causing excess iron deposition in the injured microenvironment [10, 38]. To investigate the spatial distribution and content of iron in injured spinal cord tissue, we innovatively used SR-µXRF in the study, an elemental analysis technique allowing for the examination of a very small sample with high resolution [43, 44]. In recent studies, *Ge et al.* found that sevoflurane caused iron overload in the brain by using SR-µXRF [45]. In their most recent study, Alexis N Webb *et al.* demonstrated that Se was significantly correlated with Pb in these particles in the cortex and hippocampus/ corpus callosum regions in Pb-exposed samples using the SR-µXRF [46]. However, no studies reported its application in the elemental analysis of spinal cord tissue. In the present study, a high-resolution atlas of Fe in the injured spinal cord could be visualized after SR-µXRF scanning. It also demonstrated a significant amount of iron deposition in the injured area following SCI, particularly 14 days after SCI, which was consistent with Perl's blue staining results. Our findings show that SR-µXRF is a novel and dependable technique that can be used to investigate iron distribution in the injured spinal cord with high resolution.

In addition to iron overload, we also revealed large amounts of lipids and abundant 4-hydroxynonenal, a well-known by-product of lipid peroxidation, accumulating in the injured area. Iron overload and lipid peroxidation are crucial features of cellular ferroptosis [47]. Iron overload can cause mitochondrial dysfunction during the ferroptosis process. This study also discovered ferroptosis-specific morphological changes in mitochondria over time after SCI. The Fenton reaction would eventually lead to cell death by accumulating lethal levels of lipid hydroperoxides [19, 48]. As a recently recognized form of regulated cell death, ferroptosis plays a critical role in the pathological process of SCI. In the previous study, *Yao et al. and Zhou et al.* demonstrated the iron contents, GSH levels, and the protective role of GPX4 in preventing ferroptosis [8, 14]. Meanwhile, activating the NRF2/HO-1 pathway can reduce ferroptosis following SCI by increasing GPX4 content and decreasing inflammation response [12, 14]. Studies also demonstrated that the microRNA/FSP1 axis is a crucial driver of ferroptosis in SCI [49, 50]. Even though there are a few studies that have shown that using iron chelators can inhibit ferroptosis and thus promote neurological recovery after SCI, the cellular and molecular mechanisms involved in ferroptosis are still unknown [51, 52].

We identified *Ctsb*, a driver gene of ferroptosis, is highly expressed in myeloid cells in the injured area, especially in the macrophage. Notably, the high expression region of CTSB overlaps highly with macrophage and 4-HNE, which indicates that CTSB might play a crucial role in driving the ferroptosis of macrophage during the SCI process. Transcriptomic data mining can assist us in investigating the underlying cellular and molecular mechanisms of various diseases. Through a comprehensive screening of all ferroptosis-related genes in the FerrDb, we identified the key differentially expressed genes after injury in this study. *Ctsb* expression changed dramatically among ferroptosis driver genes. CTSB is a lysosomal cysteine protease from the papain enzyme family. Under normal conditions, it is an autophagy modulator that degrades endocytosed or phagocytosed proteins. However, when lysosomal membrane permeabilization increases in inflammatory or infectious diseases, CTSB can be overactivated and released from the lysosome [53, 54]. High levels of CTSB are also found in neurodegenerative diseases, which indicates CTSB plays an important role in central nervous system diseases [55-57]. Recent research has looked into the role of CTSB in ferroptosis. *Kuang et al.* discovered that erastin-induced nuclear CTSB accumulation caused DNA damage and autophagy-dependent GPX4 degradation [58]. Most recently, *Nagakannan et al.* reported that the cytoplasmic leakage of CTSB can damage the mitochondria and induce the cleavage of nuclear histone H3. Moreover, they found CTSB is a downstream executioner of ferroptosis independent of GSH and GPX4 [59]. The released CTSB could also lead to increasing intracellular ROS through the degradation of mtTFA [34]. Our study and those above confirm that CTSB is an important molecule that could drive cellular ferroptosis. However, the role of CTSB in the ferroptosis of macrophages remains to be investigated.

Activated macrophages can be generally divided into M1 and M2 types, and the two different types of macrophages have different biological functions [7]. In this study, we found that M2-type macrophages were more sensitive to ferroptosis than M1-type macrophages. In addition, we confirmed that CA-074 could inhibit macrophage ferroptosis in vitro, particularly in M2-type macrophages. Recent research has also shown that tissue-inflammatory macrophages and alternatively activated macrophages have different ferroptosis sensitivities [36, 60]. It has been reported that the cell viability of most macrophages was unaffected in the absence of Gpx4. However, IL-4 overexpression triggered the ferroptosis of alternatively activated macrophages. Exposure to nitric oxide, in turn, restored their viability [60]. Kapralov et al. also demonstrated that activated (M1) and alternatively activated (M2) macrophages have different ferroptosis sensitivity. The enrichment of iNOS/NO^•^ - in M1 macrophages modifies their susceptibility to ferroptosis [36]. Although we confirmed that M2 is sensitive to ferroptosis, and CA-074 can inhibit ferroptosis in M2-type macrophages. The therapeutic effect of CA-074 on SCI remains to be further clarified.

The polarization state of macrophages after SCI is crucial in cell communication and remodeling of the local microenvironment [38]. Generally, M2-polarized macrophages were defined as tissue-reparative macrophages and aided tissue regeneration after SCI [61]. The intervention of macrophage activation in SCI is an efficient therapeutic strategy. Therefore, we investigated the effect of CA-074-me on SCI recovery. CA-074-me was found to induce M2 polarization after SCI by promoting the survival of M2 macrophages. Meanwhile, M2-type macrophages that survive can clean up debris in the injured microenvironment and promote nerve regeneration. CTSB in activated macrophages resulted in translocation of NF-kB and production of inflammatory cytokines [62]. CA-074-me treatment after SCI decreased the levels of IL-1β and facilitated the clearance of lipids. Timely resolution of pro-inflammatory factors and metabolic wastes from the injured area explains why inhibiting the ferroptosis of macrophages can promote the repair of SCI. Moreover, CTSB plays key roles in the activation of other myeloid cells, such as microglia. It is also an essential enzyme for the maturation and secretion of pro-inflammatory cytokines by microglia [63]. The lipid lysosomal overload in microglia induced lysosomal damage and CTSB extracellular release, which mediated neurodegeneration [64]. We showed that CTSB is also activated in microglia, and CA-074-me affected the polarization state of microglia.

It is known only the clinical application can prove the virtually effect of CA-074me on human. According to the drug information provided by Therapeutic Target Database, CA-074me is still at a preclinical stage [65]. Current studies of CA-074me have confirmed its treatment effect on fibrotic diseases, Alzheimer's disease, traumatic brain injury, ischemia, and inflammatory pain in animal models [66, 67]. Yamashima et al. applied CA-074-me to adult monkeys to treat cerebral ischemia [68]. However, due to ethical issues and the difficulty of obtaining human samples of injured spinal cords, current studies haven't verified it in human tissue. Most recently, Yadav et al. revealed the cellular taxonomy of the adult human spinal cord using single-nucleus RNA sequencing [69]. In this study, dissociated lumbar human spinal cord from seven donors without traumatic SCI was obtained. After analyzing the published data, we found that CTSB was exclusively expressed in microglia, indicating the application potential of therapies targeting macrophage/ microglia CTSB on human spinal cord injury with relatively low side effects.

To summarize, we identified that *Ctsb* is a critical ferroptosis-related gene in SCI. And our study further demonstrated that CA-074-me, a specific inhibitor for CTSB, could promote neurological function recovery by inhibiting ferroptosis in M2-type macrophages and promoting its neuroprotective function for SCI. We demonstrated that the continuous intravenous injection of CA-074-me in SCI mice was safe and effective. Despite its promising application potential, our study has some limitations. Our choice of dose and duration for the treatment is still relatively limited, and the dose-response study would be designed in future studies. Despite the fact that no other side effects were observed in the treated mice, the effect of CA-074-me on other cells has yet to be thoroughly tested. Furthermore, it should be noted that a single drug cannot completely reverse tissue damage. We will continue to investigate the role of protease and cell death in SCI and provide additional support for the efficacy of the candidate drugs.

In the present study, we elucidated that *Ctsb* is a key gene that promotes ferroptosis in macrophages. And M2-type macrophages are more susceptible to ferroptosis than M1-type macrophages. CA-074-me, a CTSB-specific inhibitor, was also shown to reduce ferroptosis, induce M2 macrophage polarization, and promote functional recovery in mice after SCI. Given the importance of ferroptosis in the progression of SCI, emerging therapies targeting CTSB may greatly facilitate treatment in the future.

## Supplementary Materials

The Supplementary data can be found online at: www.aginganddisease.org/EN/10.14336/AD.2023.0509.



## References

[1] Ahuja C S, Wilson J R, Nori S, Kotter M R N, Druschel C, Curt A, et al. (2017). Traumatic spinal cord injury. Nature reviews Disease primers, 3: 17018. 10.1038/nrdp.2017.18 28447605

[2] Baroncini A, Maffulli N, Eschweiler J, Tingart M, Migliorini F (2021). Pharmacological management of secondary spinal cord injury. Expert Opin Pharmacother, 22: 1793-1800. 33899630 10.1080/14656566.2021.1918674

[3] Dixon S J, Lemberg K M, Lamprecht M R, Skouta R, Zaitsev E M, Gleason C E, et al. (2012). Ferroptosis: an iron-dependent form of nonapoptotic cell death. Cell, 149: 1060-1072. 22632970 10.1016/j.cell.2012.03.042 PMC3367386

[4] Jiang X, Stockwell B R, Conrad M (2021). Ferroptosis: mechanisms, biology and role in disease. Nat Rev Mol Cell Biol, 22: 266-282. 33495651 10.1038/s41580-020-00324-8 PMC8142022

[5] Ahuja C S, Nori S, Tetreault L, Wilson J, Kwon B, Harrop J, et al. (2017). Traumatic Spinal Cord Injury-Repair and Regeneration. Neurosurgery, 80: S9-s22. 28350947 10.1093/neuros/nyw080

[6] Milich L M, Ryan C B, Lee J K (2019). The origin, fate, and contribution of macrophages to spinal cord injury pathology. Acta Neuropathol, 137: 785-797. 30929040 10.1007/s00401-019-01992-3 PMC6510275

[7] Hu X, Leak R K, Shi Y, Suenaga J, Gao Y, Zheng P, et al. (2015). Microglial and macrophage polarization-new prospects for brain repair. Nat Rev Neurol, 11: 56-64. 25385337 10.1038/nrneurol.2014.207 PMC4395497

[8] Yao X, Zhang Y, Hao J, Duan H Q, Zhao C X, Sun C, et al. (2019). Deferoxamine promotes recovery of traumatic spinal cord injury by inhibiting ferroptosis. Neural Regen Res, 14: 532-541. 30539824 10.4103/1673-5374.245480 PMC6334606

[9] Shi J, Tang R, Zhou Y, Xian J, Zuo C, Wang L, et al. (2020). Attenuation of White Matter Damage Following Deferoxamine Treatment in Rats After Spinal Cord Injury. World Neurosurg, 137: e9-e17. 31518742 10.1016/j.wneu.2019.08.246

[10] Ge H, Xue X, Xian J, Yuan L, Wang L, Zou Y, et al. (2022). Ferrostatin-1 Alleviates White Matter Injury Via Decreasing Ferroptosis Following Spinal Cord Injury. Mol Neurobiol, 59: 161-176. 34635980 10.1007/s12035-021-02571-y

[11] Chen Y, Liu S, Li J, Li Z, Quan J, Liu X, et al. (2020). The Latest View on the Mechanism of Ferroptosis and Its Research Progress in Spinal Cord Injury. Oxid Med Cell Longev, 2020: 6375938. 32908634 10.1155/2020/6375938 PMC7474794

[12] Gong F, Ge T, Liu J, Xiao J, Wu X, Wang H, et al. (2022). Trehalose inhibits ferroptosis via NRF2/HO-1 pathway and promotes functional recovery in mice with spinal cord injury. Aging (Albany NY), 14: 3216-3232. 35400664 10.18632/aging.204009 PMC9037257

[13] Fan B Y, Pang Y L, Li W X, Zhao C X, Zhang Y, Wang X, et al. (2021). Liproxstatin-1 is an effective inhibitor of oligodendrocyte ferroptosis induced by inhibition of glutathione peroxidase 4. Neural Regen Res, 16: 561-566. 32985488 10.4103/1673-5374.293157 PMC7996026

[14] Ge M H, Tian H, Mao L, Li D Y, Lin J Q, Hu H S, et al. (2021). Zinc attenuates ferroptosis and promotes functional recovery in contusion spinal cord injury by activating Nrf2/GPX4 defense pathway. CNS Neurosci Ther, 27: 1023-1040. 33951302 10.1111/cns.13657 PMC8339532

[15] Wu H, Wang C, Wu S (2017). Single-Cell Sequencing for Drug Discovery and Drug Development. Curr Top Med Chem, 17: 1769-1777. 27848892 10.2174/1568026617666161116145358

[16] Tica J, Bradbury E J, Didangelos A (2018). Combined Transcriptomics, Proteomics and Bioinformatics Identify Drug Targets in Spinal Cord Injury. Int J Mol Sci, 19: 1461. 29758010 10.3390/ijms19051461 PMC5983596

[17] Milich L M, Choi J S, Ryan C, Cerqueira S R, Benavides S, Yahn S L, et al. (2021). Single-cell analysis of the cellular heterogeneity and interactions in the injured mouse spinal cord. J Exp Med, 218: e20210040. 34132743 10.1084/jem.20210040 PMC8212781

[18] Zhou Y, Chen C, Guo Z, Xie S, Hu J, Lu H (2018). SR-FTIR as a tool for quantitative mapping of the content and distribution of extracellular matrix in decellularized book-shape bioscaffolds. BMC Musculoskelet Disord, 19: 220. 30021603 10.1186/s12891-018-2149-9 PMC6052527

[19] Sumneang N, Siri-Angkul N, Kumfu S, Chattipakorn S C, Chattipakorn N (2020). The effects of iron overload on mitochondrial function, mitochondrial dynamics, and ferroptosis in cardiomyocytes. Arch Biochem Biophys, 680: 108241. 31891670 10.1016/j.abb.2019.108241

[20] Bradbury E J, Burnside E R (2019). Moving beyond the glial scar for spinal cord repair. Nat Commun, 10: 3879. 31462640 10.1038/s41467-019-11707-7 PMC6713740

[21] Kyritsis N, Torres-Espín A, Schupp P G, Huie J R, Chou A, Duong-Fernandez X, et al. (2021). Diagnostic blood RNA profiles for human acute spinal cord injury. J Exp Med, 218: e20201795. 33512429 10.1084/jem.20201795 PMC7852457

[22] Satija R, Farrell J A, Gennert D, Schier A F, Regev A (2015). Spatial reconstruction of single-cell gene expression data. Nat Biotechnol, 33: 495-502. 25867923 10.1038/nbt.3192 PMC4430369

[23] Korsunsky I, Millard N, Fan J, Slowikowski K, Zhang F, Wei K, et al. (2019). Fast, sensitive and accurate integration of single-cell data with Harmony. Nat Methods, 16: 1289-1296. 31740819 10.1038/s41592-019-0619-0 PMC6884693

[24] Li C, Qin T, Zhao J, He R, Wen H, Duan C, et al. (2021). Bone Marrow Mesenchymal Stem Cell-Derived Exosome-Educated Macrophages Promote Functional Healing After Spinal Cord Injury. Front Cell Neurosci, 15: 725573. 34650405 10.3389/fncel.2021.725573 PMC8506031

[25] Park J H, Kim J H, Oh S K, Baek S R, Min J, Kim Y W, et al. (2016). Analysis of equivalent parameters of two spinal cord injury devices: the New York University impactor versus the Infinite Horizon impactor. Spine J, 16: 1392-1403. 27349631 10.1016/j.spinee.2016.06.018

[26] Chen G, Yang Y, Xu C, Gao S (2018). A Flow Cytometry-based Assay for Measuring Mitochondrial Membrane Potential in Cardiac Myocytes After Hypoxia/Reoxygenation. J Vis Exp, 13: 57725. 10.3791/57725 PMC6126459 30059023

[27] Basso D M, Fisher L C, Anderson A J, Jakeman L B, McTigue D M, Popovich P G (2006). Basso Mouse Scale for locomotion detects differences in recovery after spinal cord injury in five common mouse strains. J Neurotrauma, 23: 635-659. 16689667 10.1089/neu.2006.23.635

[28] Wang C, Zhang L, Ndong J C, Hettinghouse A, Sun G, Chen C, et al. (2019). Progranulin deficiency exacerbates spinal cord injury by promoting neuroinflammation and cell apoptosis in mice. J Neuroinflammation, 16: 238. 31775776 10.1186/s12974-019-1630-1 PMC6882111

[29] Smith R R, Burke D A, Baldini A D, Shum-Siu A, Baltzley R, Bunger M, et al. (2006). The Louisville Swim Scale: a novel assessment of hindlimb function following spinal cord injury in adult rats. J Neurotrauma, 23: 1654-1670. 17115911 10.1089/neu.2006.23.1654 PMC2833969

[30] Ni S, Luo Z, Jiang L, Guo Z, Li P, Xu X, et al. (2019). UTX/KDM6A Deletion Promotes Recovery of Spinal Cord Injury by Epigenetically Regulating Vascular Regeneration. Mol Ther, 27: 2134-2146. 31495776 10.1016/j.ymthe.2019.08.009 PMC6904668

[31] Zille M, Oses-Prieto J A, Savage S R, Karuppagounder S S, Chen Y, Kumar A, et al. (2022). Hemin-Induced Death Models Hemorrhagic Stroke and Is a Variant of Classical Neuronal Ferroptosis. J Neurosci, 42: 2065-2079. 34987108 10.1523/JNEUROSCI.0923-20.2021 PMC8916756

[32] Xu Y, Wang J, Song X, Wei R, He F, Peng G, et al. (2016). Protective mechanisms of CA074-me (other than cathepsin-B inhibition) against programmed necrosis induced by global cerebral ischemia/reperfusion injury in rats. Brain Res Bull, 120: 97-105. 26562519 10.1016/j.brainresbull.2015.11.007

[33] Yamamoto A, Tomoo K, Hara T, Murata M, Kitamura K, Ishida T (2000). Substrate specificity of bovine cathepsin B and its inhibition by CA074, based on crystal structure refinement of the complex. J Biochem, 127: 635-643. 10739956 10.1093/oxfordjournals.jbchem.a022651

[34] Wu Z, Han X, Bao J, Li B, Shen J, Song P, et al. (2022). Dopamine D2 Receptor Signaling Attenuates Acinar Cell Necroptosis in Acute Pancreatitis through the Cathepsin B/TFAM/ROS Pathway. Oxid Med Cell Longev, 2022: 4499219. 35927992 10.1155/2022/4499219 PMC9345736

[35] Doll S, Proneth B, Tyurina Y Y, Panzilius E, Kobayashi S, Ingold I, et al. (2017). ACSL4 dictates ferroptosis sensitivity by shaping cellular lipid composition. Nat Chem Biol, 13: 91-98. 27842070 10.1038/nchembio.2239 PMC5610546

[36] Kapralov A A, Yang Q, Dar H H, Tyurina Y Y, Anthonymuthu T S, Kim R, et al. (2020). Redox lipid reprogramming commands susceptibility of macrophages and microglia to ferroptotic death. Nat Chem Biol, 16: 278-290. 32080625 10.1038/s41589-019-0462-8 PMC7233108

[37] Li Y, He X, Kawaguchi R, Zhang Y, Wang Q, Monavarfeshani A, et al. (2020). Microglia-organized scar-free spinal cord repair in neonatal mice. Nature, 587: 613-618. 33029008 10.1038/s41586-020-2795-6 PMC7704837

[38] Ge X, Tang P, Rong Y, Jiang D, Lu X, Ji C, et al. (2021). Exosomal miR-155 from M1-polarized macrophages promotes EndoMT and impairs mitochondrial function via activating NF-κB signaling pathway in vascular endothelial cells after traumatic spinal cord injury. Redox biology, 41: 101932. 33714739 10.1016/j.redox.2021.101932 PMC7967037

[39] Luo Z, Peng W, Xu Y, Xie Y, Liu Y, Lu H, et al. (2021). Exosomal OTULIN from M2 macrophages promotes the recovery of spinal cord injuries via stimulating Wnt/β-catenin pathway-mediated vascular regeneration. Acta biomaterialia, 136: 519-532. 34551329 10.1016/j.actbio.2021.09.026

[40] Yunna C, Mengru H, Lei W, Weidong C (2020). Macrophage M1/M2 polarization. European journal of pharmacology, 877: 173090. 32234529 10.1016/j.ejphar.2020.173090

[41] Zhang T, Xu W, Lin X, Yan H, Ma M, He Z (2019). Assessment of heavy metals pollution of soybean grains in North Anhui of China. Sci Total Environ, 646: 914-922. 30067961 10.1016/j.scitotenv.2018.07.335

[42] Hu X, Leak R K, Thomson A W, Yu F, Xia Y, Wechsler L R, et al. (2018). Promises and limitations of immune cell-based therapies in neurological disorders. Nat Rev Neurol, 14: 559-568. 29925925 10.1038/s41582-018-0028-5 PMC6237550

[43] Wang M, Zuo Y, Li X, Li Y, Thirupathi A, Yu P, et al. (2021). Effect of sevoflurane on iron homeostasis and toxicity in the brain of mice. Brain Res, 1757: 147328. 33539795 10.1016/j.brainres.2021.147328

[44] Al-Sandaqchi A T, Brignell C, Collingwood J F, Geraki K, Mirkes E M, Kong K, et al. (2018). Metallome of cerebrovascular endothelial cells infected with Toxoplasma gondii using mu-XRF imaging and inductively coupled plasma mass spectrometry. Metallomics, 10: 1401-1414. 30183049 10.1039/c8mt00136g

[45] Ge X, Zuo Y, Xie J, Li X, Li Y, Thirupathi A, et al. (2021). A new mechanism of POCD caused by sevoflurane in mice: cognitive impairment induced by cross-dysfunction of iron and glucose metabolism. Aging (Albany NY), 13: 22375-22389. 34547719 10.18632/aging.203544 PMC8507282

[46] Webb A N, Spiers K M, Falkenberg G, Gu H, Dwibhashyam S S, Du Y, et al. (2022). Distribution of Pb and Se in mouse brain following subchronic Pb exposure by using synchrotron X-ray fluorescence. Neurotoxicology, 88: 106-115. 34793780 10.1016/j.neuro.2021.11.006 PMC8748384

[47] Zheng J, Conrad M (2020). The Metabolic Underpinnings of Ferroptosis. Cell Metab, 32: 920-937. 33217331 10.1016/j.cmet.2020.10.011

[48] Stockwell B R, Friedmann Angeli J P, Bayir H, Bush A I, Conrad M, Dixon S J, et al. (2017). Ferroptosis: A Regulated Cell Death Nexus Linking Metabolism, Redox Biology, and Disease. Cell, 171: 273-285. 28985560 10.1016/j.cell.2017.09.021 PMC5685180

[49] Shao C, Chen Y, Yang T, Zhao H, Li D (2022). Mesenchymal Stem Cell Derived Exosomes Suppress Neuronal Cell Ferroptosis Via lncGm36569/miR-5627-5p/FSP1 Axis in Acute Spinal Cord Injury. Stem Cell Rev Rep, 18: 1127-1142. 35257299 10.1007/s12015-022-10327-x

[50] Wang F, Li J, Zhao Y, Guo D, Liu D, Chang S, et al. (2022). miR-672-3p Promotes Functional Recovery in Rats with Contusive Spinal Cord Injury by Inhibiting Ferroptosis Suppressor Protein 1. Oxid Med Cell Longev, 2022: 6041612. 35237382 10.1155/2022/6041612 PMC8885177

[51] Feng Z, Min L, Chen H, Deng W, Tan M, Liu H, et al. (2021). Iron overload in the motor cortex induces neuronal ferroptosis following spinal cord injury. Redox biology, 43: 101984. 33933882 10.1016/j.redox.2021.101984 PMC8105676

[52] Zhang Y, Fan B Y, Pang Y L, Shen W Y, Wang X, Zhao C X, et al. (2020). Neuroprotective effect of deferoxamine on erastininduced ferroptosis in primary cortical neurons. Neural Regen Res, 15: 1539-1545. 31997820 10.4103/1673-5374.274344 PMC7059591

[53] Mort J S, Buttle D J (1997). Cathepsin B. Int J Biochem Cell Biol, 29: 715-720. 9251238 10.1016/s1357-2725(96)00152-5

[54] Chen X, Yu C, Kang R, Kroemer G, Tang D (2021). Cellular degradation systems in ferroptosis. Cell Death Differ, 28: 1135-1148. 33462411 10.1038/s41418-020-00728-1 PMC8027807

[55] Mijanović O, Branković A, Panin A N, Savchuk S, Timashev P, Ulasov I, et al. (2019). Cathepsin B: A sellsword of cancer progression. Cancer Lett, 449: 207-214. 30796968 10.1016/j.canlet.2019.02.035 PMC6488514

[56] Liu Y, Xue X, Zhang H, Che X, Luo J, Wang P, et al. (2019). Neuronal-targeted TFEB rescues dysfunction of the autophagy-lysosomal pathway and alleviates ischemic injury in permanent cerebral ischemia. Autophagy, 15: 493-509. 30304977 10.1080/15548627.2018.1531196 PMC6351122

[57] Bellomo G, Paciotti S, Gatticchi L, Parnetti L (2020). The vicious cycle between alpha-synuclein aggregation and autophagic-lysosomal dysfunction. Mov Disord, 35: 34-44. 31729779 10.1002/mds.27895

[58] Kuang F, Liu J, Li C, Kang R, Tang D (2020). Cathepsin B is a mediator of organelle-specific initiation of ferroptosis. Biochem Biophys Res Commun, 533: 1464-1469. 33268027 10.1016/j.bbrc.2020.10.035

[59] Nagakannan P, Islam M I, Conrad M, Eftekharpour E (2021). Cathepsin B is an executioner of ferroptosis. Biochim Biophys Acta Mol Cell Res, 1868: 118928. 33340545 10.1016/j.bbamcr.2020.118928

[60] Piattini F, Matsushita M, Muri J, Bretscher P, Feng X, Freigang S, et al. (2021). Differential sensitivity of inflammatory macrophages and alternatively activated macrophages to ferroptosis. European journal of immunology, 51: 2417-2429. 34272880 10.1002/eji.202049114 PMC9290640

[61] Gensel J C, Zhang B (2015). Macrophage activation and its role in repair and pathology after spinal cord injury. Brain Res, 1619: 1-11. 25578260 10.1016/j.brainres.2014.12.045

[62] Sendler M, Weiss F U, Golchert J, Homuth G, van den Brandt C, Mahajan U M, et al. (2018). Cathepsin B-Mediated Activation of Trypsinogen in Endocytosing Macrophages Increases Severity of Pancreatitis in Mice. Gastroenterology, 154: 704-718.e710. 29079517 10.1053/j.gastro.2017.10.018 PMC6663074

[63] Sun L, Wu Z, Hayashi Y, Peters C, Tsuda M, Inoue K, et al. (2012). Microglial cathepsin B contributes to the initiation of peripheral inflammation-induced chronic pain. J Neurosci, 32: 11330-11342. 22895716 10.1523/JNEUROSCI.0677-12.2012 PMC6621185

[64] Gabandé-Rodríguez E, Pérez-Cañamás A, Soto-Huelin B, Mitroi D N, Sánchez-Redondo S, Martínez-Sáez E, et al. (2019). Lipid-induced lysosomal damage after demyelination corrupts microglia protective function in lysosomal storage disorders. The EMBO journal, 38: e99553. 30530526 10.15252/embj.201899553 PMC6331723

[65] Zhou Y, Zhang Y, Lian X, Li F, Wang C, Zhu F, et al. (2022). Therapeutic target database update 2022: facilitating drug discovery with enriched comparative data of targeted agents. Nucleic acids research, 50: D1398-d1407. 34718717 10.1093/nar/gkab953 PMC8728281

[66] Li X, Zhu L, Wang B, Yuan M, Zhu R (2017). Drugs and Targets in Fibrosis. Frontiers in pharmacology, 8: 855. 29218009 10.3389/fphar.2017.00855 PMC5703866

[67] Hook V, Yoon M, Mosier C, Ito G, Podvin S, Head B P, et al. (2020). Cathepsin B in neurodegeneration of Alzheimer's disease, traumatic brain injury, and related brain disorders. Biochimica et biophysica acta Proteins and proteomics, 1868: 140428. 32305689 10.1016/j.bbapap.2020.140428 PMC7261628

[68] Yamashima T, Kohda Y, Tsuchiya K, Ueno T, Yamashita J, Yoshioka T, et al. (1998). Inhibition of ischaemic hippocampal neuronal death in primates with cathepsin B inhibitor CA-074: a novel strategy for neuroprotection based on 'calpain-cathepsin hypothesis'. The European journal of neuroscience, 10: 1723-1733. 9751144 10.1046/j.1460-9568.1998.00184.x

[69] Yadav A, Matson K J E, Li L, Hua I, Petrescu J, Kang K, et al. (2023). A cellular taxonomy of the adult human spinal cord. Neuron, 111: 328-344.e327. 36731429 10.1016/j.neuron.2023.01.007 PMC10044516

