# Supplementary material for: Identification of Cathepsin B as a Therapeutic Target for Ferroptosis of Macrophage after Spinal Cord Injury
Source: Aging Dis. 2024 Feb 1;15(1):421–43. doi: 10.14336/AD.2023.0509 (PMC10796092; doi:10.14336/AD.2023.0509)
Supplement: Supplementary file 1 [file AD-15-1-421-s.pdf]

SUPPLEMENTARY DATA

Identification of Cathepsin B as a Therapeutic Target for Ferroptosis of Macrophage after Spinal Cord Injury

Jiaqi Xu, Yinghe Ding, Chaoran Shi, Feifei Yuan, Xiaolong Sheng, Yudong Liu, Yong Xie, Hongbin Lu, Chunyue Duan, Jianzhong Hu, Liyuan Jiang

© 2023. Xu J et al. Published online at http://www.aginganddisease.org/EN/10.14336/AD.2023.0509

SUPPLEMENTARY DATA

Supplementary Methods

RNA isolation and quantitative real-time PCR

Approximately 0.5 cm of spinal cord tissue around the injured epicenter was harvested. For in vitro experiments, the cells that were seeded in the six-well plates were harvested. RNA was extracted using TRIzol reagent (Invitrogen). Reverse transcription and qRT-PCR were performed using the GoScript™ Reverse Transcription System and the qPCR Master Mix (Promega), following the manufacturer's instructions. All reactions were processed and analyzed on an ABI 7900 fast real-time PCR system (Applied Biosystems). The relative expression levels of the target genes were normalized to GAPDH using the $2^{-\Delta\Delta CT}$ method.

Primers used are listed:

Gene	Forward primer (5' to 3')	Reverse primer (5' to 3')
**GAPDH**	AGGTCGGTGTGAACGGATTTG	TGTAGACCATGTAGTTGAGGTCA
**Ctsb**	TCCTTGATCCTTCTTTCTTGCC	ACAGTGCCACACAGCTTCTTC

Enzyme-Linked Immunosorbent Assay (ELISA) of CTSB

Approximately, 0.5 cm of spinal cord tissue around the injured epicenter was harvested. After the tissue was cut, homogenized, and centrifuged, the supernatant was collected. The content of mouse CTSB was measured and calculated using the Mouse CTSB (Cathepsin B) ELISA kit (Elabscience Biotechnology, E-EL-M2423c), following the manufacturer's instructions.

© 2023. Xu J et al. Published online at http://www.aginganddisease.org/EN/10.14336/AD.2023.0509

SUPPLEMENTARY DATA

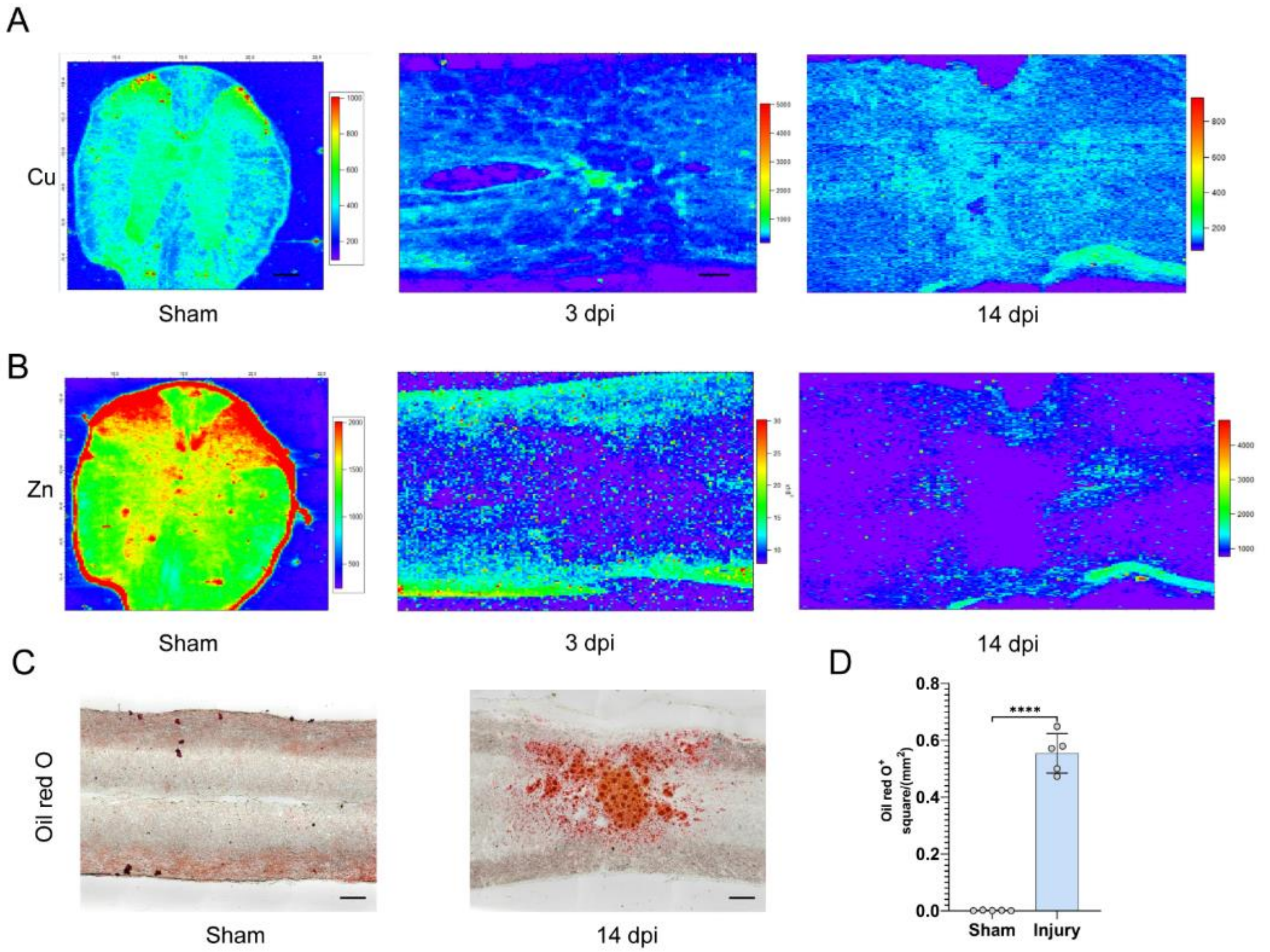

Supplementary Figure 1. (A) SRµXRF image of the content of copper in the spinal cord in sham group, and at 3 dpi and 14 dpi. (B) SRµXRF image of the content of zinc in the spinal cord in sham group, and at 3 dpi and 14 dpi. (C) Oil red O staining of the lipid content in the sham group and at 14 dpi. (D) Quantification of the Oil red O square in (C) (n=5, mean ± SD, Welch's test, **** $p < 0.0001$). (Scale bar = 200 µm)

© 2023. Xu J et al. Published online at http://www.aginganddisease.org/EN/10.14336/AD.2023.0509

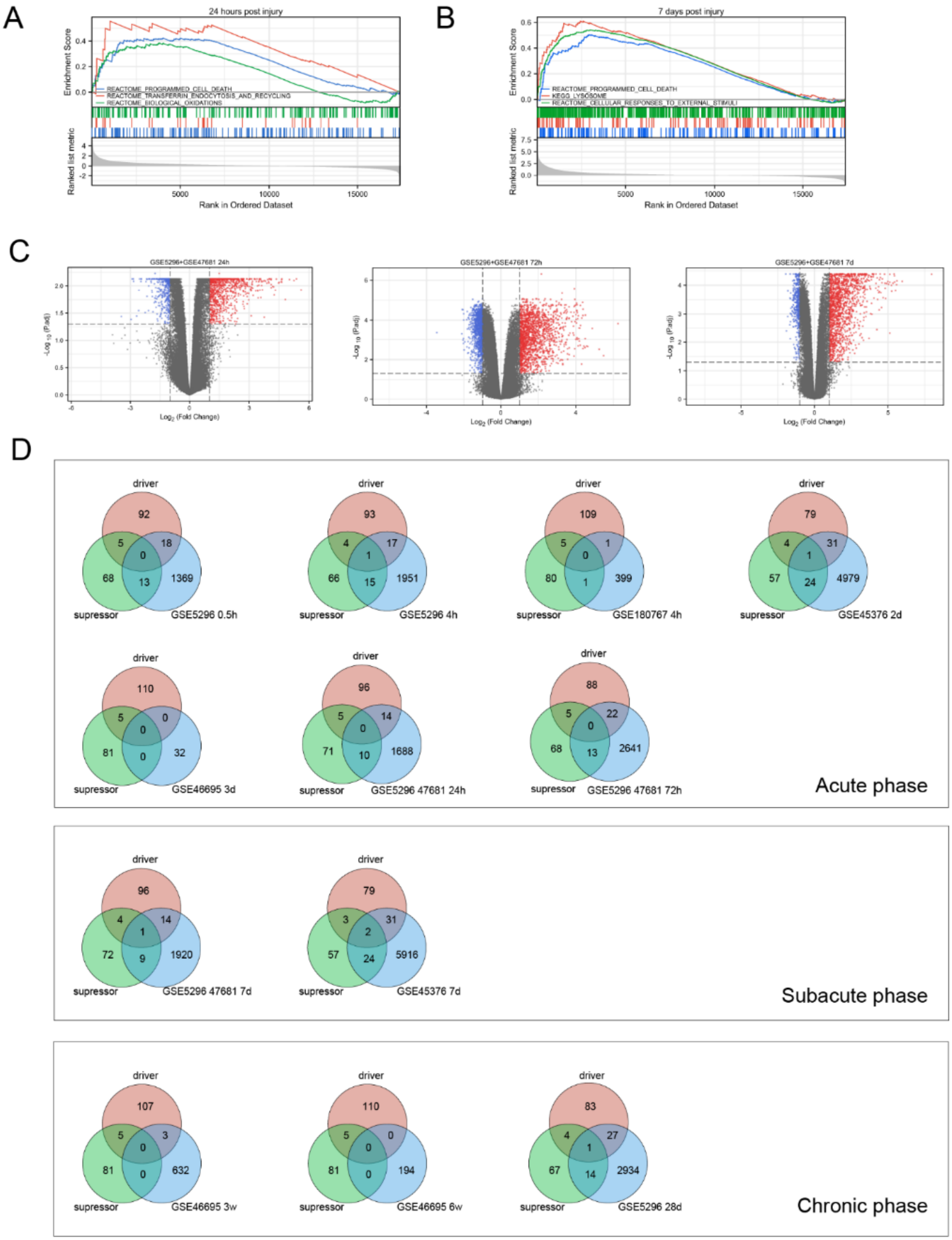

Supplementary Figure 2. (A) GSEA visualization of pathways enriched in GSE5296 and GSE47681 at 24 hpi (injury vs sham). (B) GSEA visualization of pathways enriched in GSE5296 and GSE47681 at 7 dpi (injury vs sham). (C) Volcano plot showing the distributions of differentially expressed genes (DEGs) in the combined dataset (GSE5296 and GSE47681) at 24 hpi, 72 hpi, and 7 dpi. (D) Venn plot showing the number of differentially expressed ferroptosis driver/suppressor genes in DEGs of different datasets.

© 2023. Xu J et al. Published online at http://www.aginganddisease.org/EN/10.14336/AD.2023.0509

SUPPLEMENTARY DATA

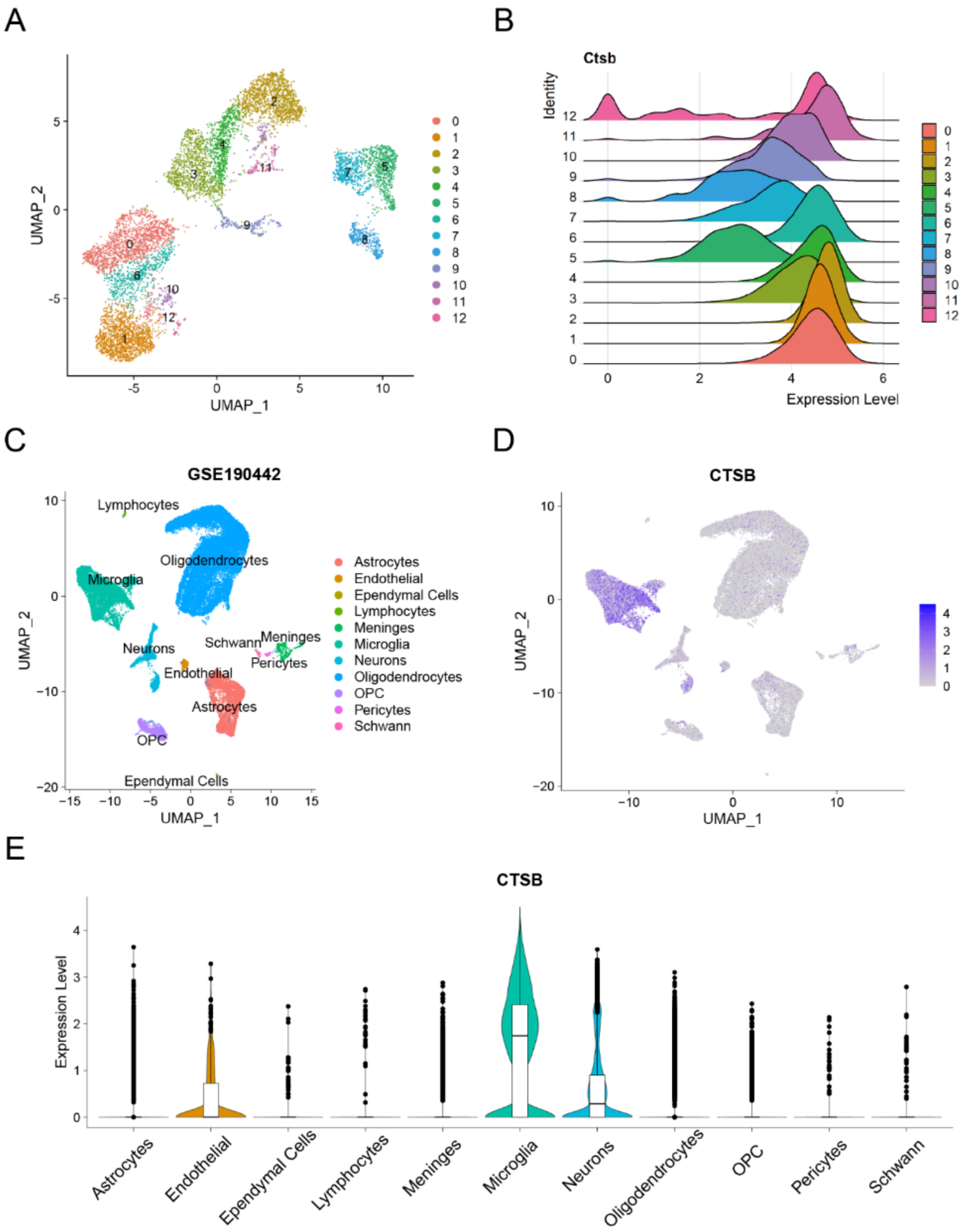

Supplementary Figure 3. (A) UMAP plot showing calculated 12 cell clusters of macrophages (GSE162610). (B) Ridge plot showing the expression level of *Ctsb* in 12 macrophage clusters (GSE162610). (C) UMAP plot showing annotated celltypes in adult human spinal cord (GSE190442). (D) Scatter plot showing the expression level of *Ctsb* in different celltypes (GSE190442). (E) Violin plot showing the expression level of *Ctsb* in different celltypes (GSE190442).

© 2023. Xu J et al. Published online at http://www.aginganddisease.org/EN/10.14336/AD.2023.0509

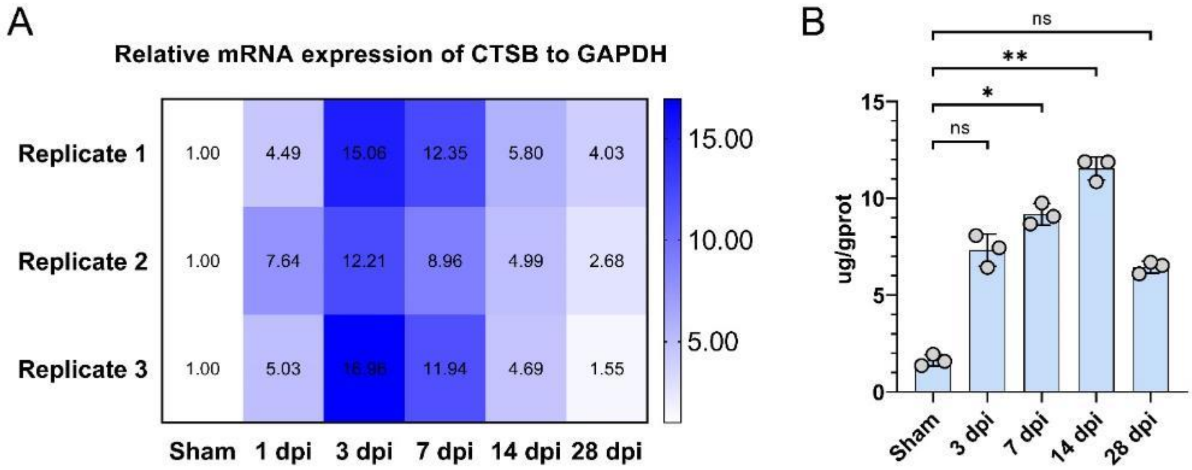

Supplementary Figure 4. (A) The relative gene expression of *Ctsb* after SCI detected by qRT-PCR. (B) The content of CTSB in the spinal cord tissue after SCI detected by ELISA (n=3, median with range, Kruskal-Wallis test, Dunn's multiple comparisons, ns not significant, * $p < 0.01$, ** $p < 0.001$).

© 2023. Xu J et al. Published online at http://www.aginganddisease.org/EN/10.14336/AD.2023.0509

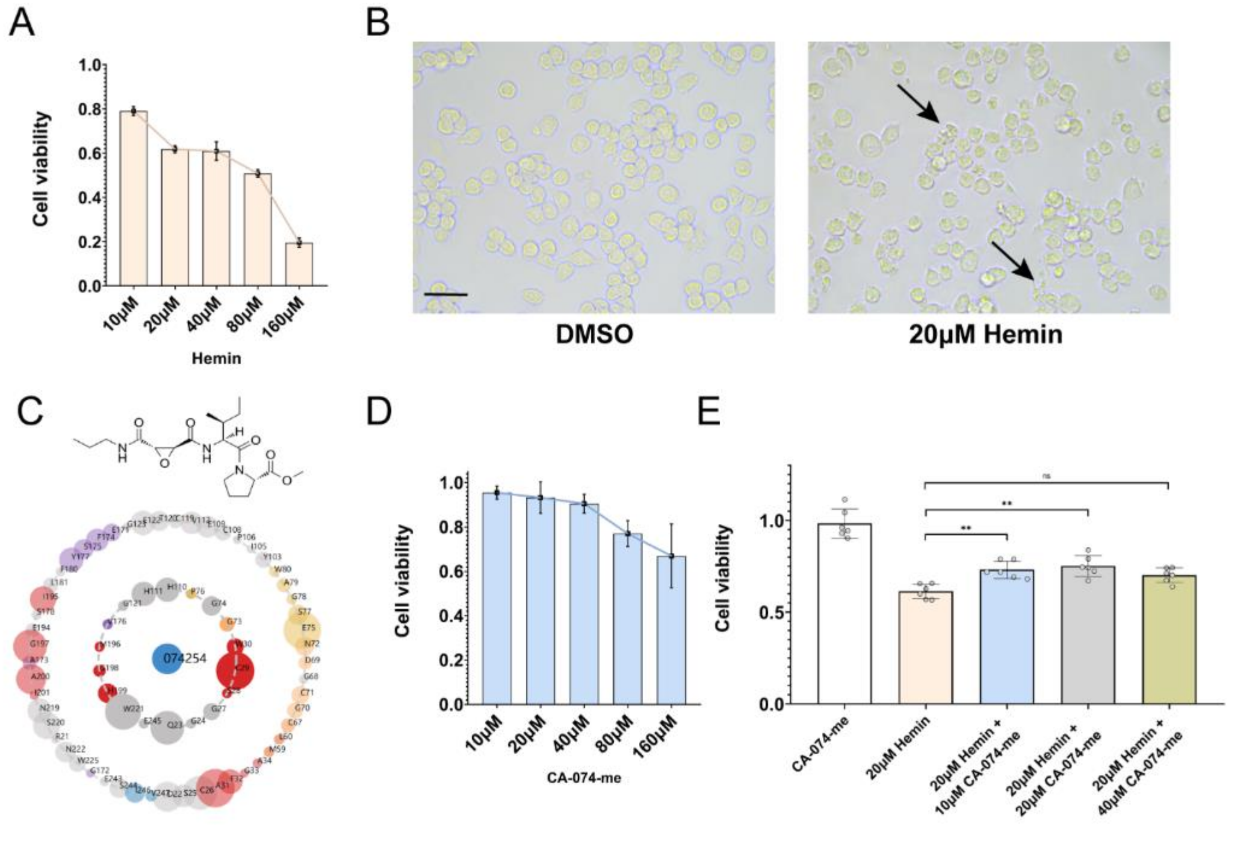

Supplementary Figure 5. (A) Cell viability with different concentrations of hemin. (n=5, mean ± SD, one-way ANOVA, p<0.0001) (B) Cells observed under an inverted optical microscope before and after hemin exposure. The black arrow indicated the rupture of the cell membrane. (Scale bar = 50 µm) (C) Ligands and residues plot created with the Protein Contacts Atlas. The inner ring indicated immediate atomic contacts. The outer ring indicated extended atomic contacts. Size of the circle is proportional to the total number of contacts the residue is involved. Colors indicated the secondary structure of residues. The plots indicated the immediate atomic contacts with amino acids, including Gln23, Gly24, Gly27, Cys29, Gly74, Pro76, His110, His111, Val176, Met196, Gly198, His199, Trp221, and Glu245. (D) Cell viability with different concentrations of CA-074-me. (n=5, mean ± SD, one-way ANOVA, p<0.0001) (E) Cell viability with different treatments of CA-074-me and/or hemin. (n=6, mean ± SD, one-way ANOVA, Tukey's multiple comparisons, ns not significant, ** p < 0.01)

© 2023. Xu J et al. Published online at http://www.aginganddisease.org/EN/10.14336/AD.2023.0509

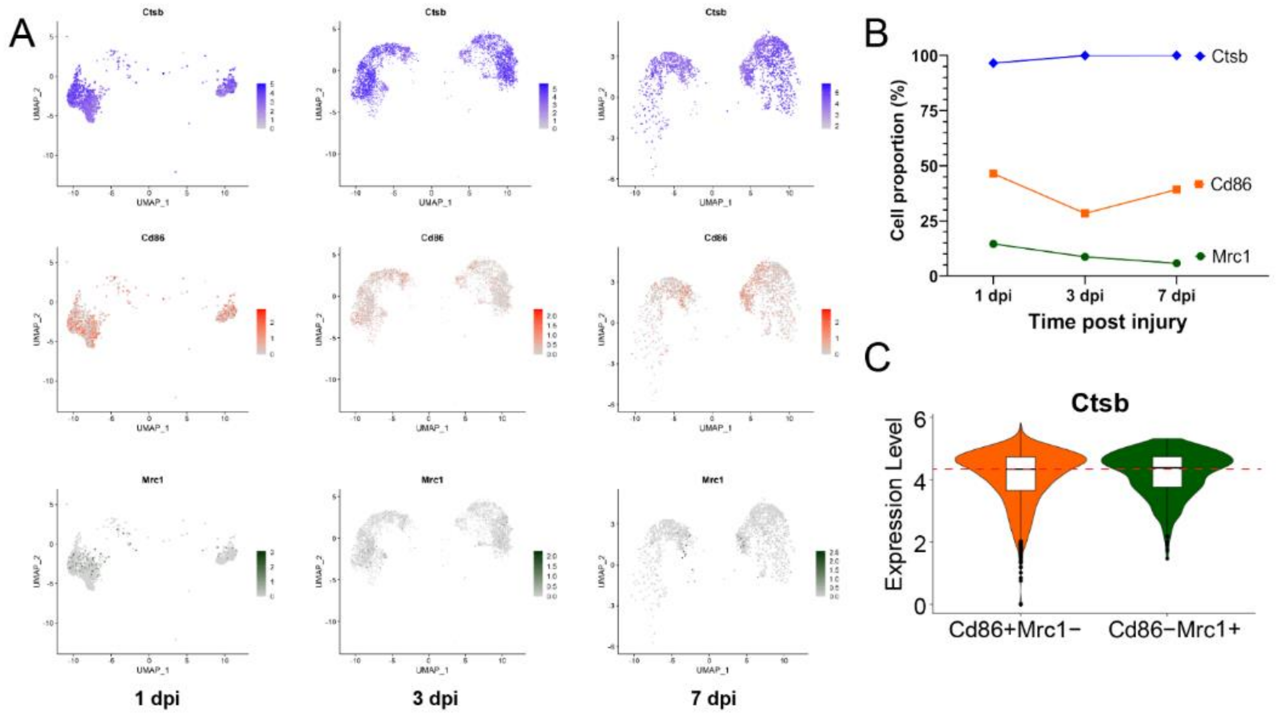

Supplementary Figure 6. (A) UMAP plot showing the expression of *Ctsb*, *Cd86*, and *Mrc1* in macrophages after SCI. (B) The calculated cell proportion of CTSB, CD86, and Mrc1 positive cells in macrophages after SCI. (C) The expression level of *Ctsb* in *Cd86*$^{+}$ *Mrc1*$^{-}$ macrophages and *Cd86*$^{-}$ *Mrc1*$^{+}$ macrophages (GSE162610).

© 2023. Xu J et al. Published online at http://www.aginganddisease.org/EN/10.14336/AD.2023.0509

SUPPLEMENTARY DATA

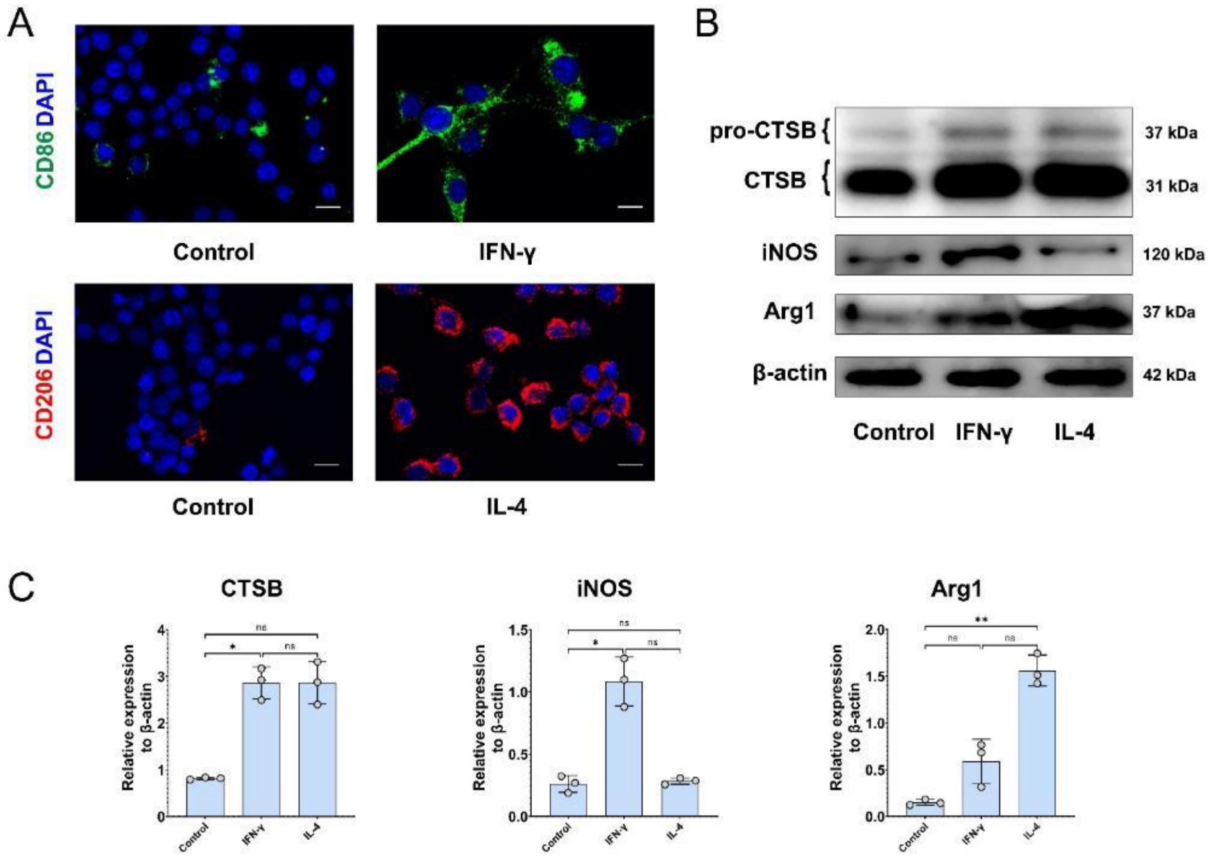

Supplementary Figure 7. (A) Representative immunofluorescence image of CD86 (green) or CD206 (red), and nucleus (blue) in IFN-γ or IL-4 induced RAW 264.7 (Scale bar = 10 μm). (B) Western blotting analysis of the levels of CTSB, iNOS, Arg1, and β-actin in IFN-γ or IL-4 induced RAW 264.7. (C) Quantification of the relative expression of CTSB, iNOS, and Arg1 to β-actin in (B) (n=3, median with range, Kruskal-Wallis test, Dunn's multiple comparisons, ns not significant, $* p < 0.05$, $** p < 0.01$).

© 2023. Xu J et al. Published online at http://www.aginganddisease.org/EN/10.14336/AD.2023.0509

SUPPLEMENTARY DATA

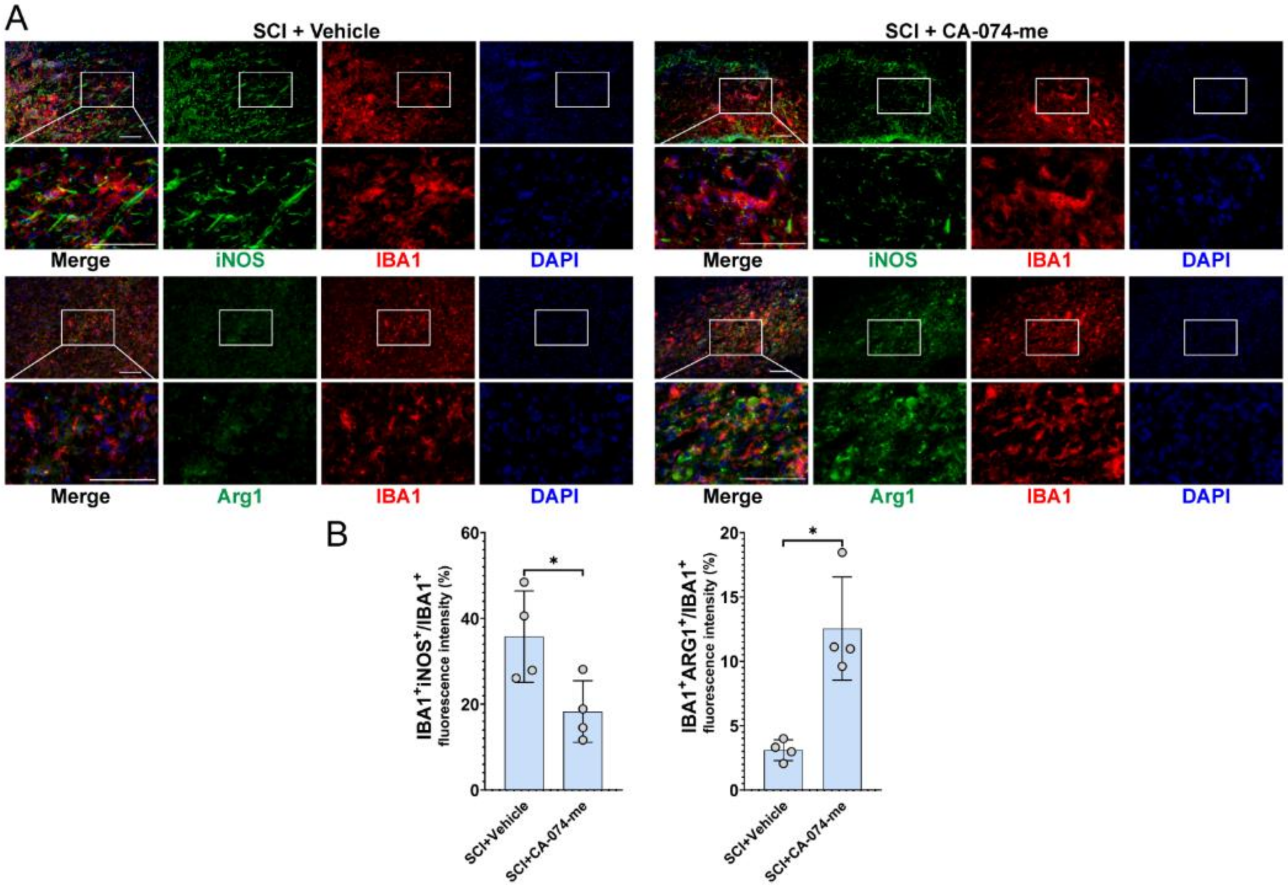

Supplementary Figure 8. (A) Representative immunofluorescence image of iNOS or Arg1 (green), IBA1 (red), and nucleus (blue) in the injured spinal cord at 14 dpi. (Scale bar = 200 μm) (B) Quantification of the $IBA1^+iNOS^+/IBA1^+$(unpaired t test) and $IBA1^+Arg1^+/IBA1^+$(Welch's test) fluorescence intensity in (A) (n=4, mean ± SD, ∗ $p < 0.05$).

© 2023. Xu J et al. Published online at http://www.aginganddisease.org/EN/10.14336/AD.2023.0509

SUPPLEMENTARY DATA

Supplementary Table 1. Ferroptosis driver/suppressor genes tested in mice from the FerrDb

	symbol	hgncid	ensgstable	testin	confidence	
1	PIK3CA	HGNC:8975	ENSG00000121879	Mice	Validated	driver
2	FLT3	HGNC:3765	ENSG00000122025	Mice	Validated	driver
3	SCP2	HGNC:10606	ENSG00000116171	Mice	Validated	driver
4	TP53	HGNC:11998	ENSG00000141510	Human, mice	Validated	driver
5	TF	HGNC:11740	ENSG00000091513	Mice	Validated	driver
6	TFRC	HGNC:11763	ENSG00000072274	Mice	Validated	driver
7	TFR2	HGNC:11762	ENSG00000106327	Mice	Validated	driver
8	SLC38A1	HGNC:13447	ENSG00000111371	Mice	Validated	driver
9	SLC1A5	HGNC:10943	ENSG00000105281	Mice	Validated	driver
10	GLS2	HGNC:29570	ENSG00000135423	Mice	Validated	driver
11	GOT1	HGNC:4432	ENSG00000120053	Mice	Validated	driver
12	ALOX5	HGNC:435	ENSG00000012779	Mice	Validated	driver
13	KEAP1	HGNC:23177	ENSG00000079999	Human, mice	Validated	driver
14	HMOX1	HGNC:5013	ENSG00000100292	Human, mice	Validated	driver
15	ATG5	HGNC:589	ENSG00000057663	Human, mice	Validated	driver
16	ATG7	HGNC:16935	ENSG00000197548	Human, mice	Validated	driver
17	ACO1	HGNC:117	ENSG00000122729	Mice	Screened	driver
18	IREB2	HGNC:6115	ENSG00000136381	Mice	Screened	driver
19	G6PDX	_NA_	_NA_	Mice	Screened	driver
20	ULK1	HGNC:12558	ENSG00000177169	Mice	Validated	driver
21	ATG3	HGNC:20962	ENSG00000144848	Mice	Validated	driver
22	ATG4D	HGNC:20789	ENSG00000130734	Mice	Screened	driver
23	BECN1	HGNC:1034	ENSG00000126581	Mice	Screened	driver
24	MAP1LC3A	HGNC:6838	ENSG00000101460	Mice	Screened	driver
25	GABARAPL2	HGNC:13291	ENSG00000034713	Mice	Screened	driver
26	GABARAPL1	HGNC:4068	ENSG00000139112	Mice	Screened	driver
27	ATG16L1	HGNC:21498	ENSG00000085978	Mice	Screened	driver
28	WIPI1	HGNC:25471	ENSG00000070540	Mice	Screened	driver
29	WIPI2	HGNC:32225	ENSG00000157954	Mice	Screened	driver
30	SNX4	HGNC:11175	ENSG00000114520	Mice	Screened	driver
31	ATG13	HGNC:29091	ENSG00000175224	Mice	Validated	driver
32	ULK2	HGNC:13480	ENSG00000083290	Mice	Validated	driver
33	NCOA4	HGNC:7671	ENSG00000266412	Human, mice	Validated	driver
34	SAT1	HGNC:10540	ENSG00000130066	Human, mice	Validated	driver
35	ACSL4	HGNC:3571	ENSG00000068366	Mice	Validated	driver
36	LPCAT3	HGNC:30244	ENSG00000111684	Mice	Validated	driver
37	ALOX15	HGNC:433	ENSG00000161905	Mice	Validated	driver
38	BID	HGNC:1050	ENSG00000015475	Mice	Validated	driver
39	DPP4	HGNC:3009	ENSG00000197635	Human, mice	Validated	driver
40	CDKN2A	HGNC:1787	ENSG00000147889	Human, mice	Validated	driver
41	PEBP1	HGNC:8630	ENSG00000089220	Human, mice	Validated	driver
42	MAPK14	HGNC:6876	ENSG00000112062	Mice	Validated	driver

© 2023. Xu J et al. Published online at http://www.aginganddisease.org/EN/10.14336/AD.2023.0509

43	ELAVL1	HGNC:3312	ENSG00000066044	Human, mice	Validated	driver
44	EPAS1	HGNC:3374	ENSG00000116016	Mice	Validated	driver
45	HILPDA	HGNC:28859	ENSG00000135245	Mice	Validated	driver
46	HIF1A	HGNC:4910	ENSG00000100644	Mice	Validated	driver
47	ALOX12	HGNC:429	ENSG00000108839	Human, mice	Validated	driver
48	IFNG	HGNC:5438	ENSG00000111537	Human, mice	Validated	driver
49	ANO6	HGNC:25240	ENSG00000177119	Human, mice	Validated	driver
50	LPIN1	HGNC:13345	ENSG00000134324	Mice	Validated	driver
51	HMGB1	HGNC:4983	ENSG00000189403	Human, mice	Validated	driver
52	YY1AP1	HGNC:30935	ENSG00000163374	Human, mice	Validated	driver
53	EGLN2	HGNC:14660	ENSG00000269858	Human, mice	Validated	driver
54	MIOX	HGNC:14522	ENSG00000100253	Human, mice	Validated	driver
55	TAFAZZIN	HGNC:11577	ENSG00000102125	Human, mice	Validated	driver
56	MTDH	HGNC:29608	ENSG00000147649	Human, mice	Validated	driver
57	SIRT1	HGNC:14929	ENSG00000096717	Mice	Predicted	driver
58	FBXW7	HGNC:16712	ENSG00000109670	Human, mice	Deduced	driver
59	PANX1	HGNC:8599	ENSG00000110218	Human, mice	Validated	driver
60	DNAJB6	HGNC:14888	ENSG00000105993	Human, mice	Predicted	driver
61	BACH1	HGNC:935	ENSG00000156273	Mice	Validated	driver
62	IL1B	HGNC:5992	ENSG00000125538	Human, mice	Validated	driver
63	POR	HGNC:9208	ENSG00000127948	Human, mice	Validated	driver
64	NR1D1	HGNC:7962	ENSG00000126368	Mice	Deduced	driver
65	NR1D2	HGNC:7963	ENSG00000174738	Mice	Deduced	driver
66	TBK1	HGNC:11584	ENSG00000183735	Mice	Validated	driver
67	IL6	HGNC:6018	ENSG00000136244	Human, mice	Validated	driver
68	CTSB	HGNC:2527	ENSG00000164733	Human, mice	Deduced	driver
69	ATF4	HGNC:786	ENSG00000128272	Human, mice	Validated	driver
70	LINC00618	HGNC:20110	ENSG00000225163	Human, mice	Validated	driver
71	PEX10	HGNC:8851	ENSG00000157911	Human, mice	Validated	driver
72	LIG3	HGNC:6600	ENSG00000005156	Human, mice	Screened	driver
73	ZEB1	HGNC:11642	ENSG00000148516	Human, mice	Validated	driver
74	PVT1	HGNC:9709	ENSG00000249859	Human, mice	Validated	driver
75	SLC39A14	HGNC:20858	ENSG00000104635	Mice	Validated	driver
76	MAP3K11	HGNC:6850	ENSG00000173327	Mice	Validated	driver
77	BRD7	HGNC:14310	ENSG00000166164	Human, mice	Validated	driver
78	SLC25A28	HGNC:23472	ENSG00000155287	Human, mice	Validated	driver
79	TSC1	HGNC:12362	ENSG00000165699	Human, mice	Validated	driver
80	LGMN	HGNC:9472	ENSG00000100600	Mice	Validated	driver
81	IFNA1	HGNC:5417	ENSG00000197919	Human, mice	Deduced	driver
82	IFNA2	HGNC:5423	ENSG00000188379	Human, mice	Deduced	driver
83	IFNA4	HGNC:5425	ENSG00000236637	Human, mice	Deduced	driver
84	IFNA5	HGNC:5426	ENSG00000147873	Human, mice	Deduced	driver
85	IFNA6	HGNC:5427	ENSG00000120235	Human, mice	Deduced	driver
86	IFNA7	HGNC:5428	ENSG00000214042	Human, mice	Deduced	driver
87	IFNA8	HGNC:5429	ENSG00000120242	Human, mice	Deduced	driver

© 2023. Xu J et al. Published online at http://www.aginganddisease.org/EN/10.14336/AD.2023.0509

88	IFNA10	HGNC:5418	ENSG00000186803	Human, mice	Deduced	driver
89	IFNA13	HGNC:5419	ENSG00000233816	Human, mice	Deduced	driver
90	IFNA14	HGNC:5420	ENSG00000228083	Human, mice	Deduced	driver
91	IFNA16	HGNC:5421	ENSG00000147885	Human, mice	Deduced	driver
92	IFNA17	HGNC:5422	ENSG00000234829	Human, mice	Deduced	driver
93	IFNA21	HGNC:5424	ENSG00000137080	Human, mice	Deduced	driver
94	PPARG	HGNC:9236	ENSG00000132170	Mice	Validated	driver
95	NOX4	HGNC:7891	ENSG00000086991	Mice	Validated	driver
96	MAP3K14	HGNC:6853	ENSG00000006062	Mice	Validated	driver
97	CircKDM4C	_NA_	_NA_	Human, mice	Validated	driver
98	MIB2	HGNC:30577	ENSG00000197530	Mice	Validated	driver
99	TRIM21	HGNC:11312	ENSG00000132109	Mice	Validated	driver
100	DPEP1	HGNC:3002	ENSG00000015413	Mice	Validated	driver
101	IDO1	HGNC:6059	ENSG00000131203	Mice	Validated	driver
102	GJA1	HGNC:4274	ENSG00000152661	Human, mice	Validated	driver
103	PGRMC1	HGNC:16090	ENSG00000101856	Mice	Validated	driver
104	USP11	HGNC:12609	ENSG00000102226	Mice	Validated	driver
105	ZFAS1	HGNC:33101	ENSG00000177410	Mice	Validated	driver
106	PIEZO1	HGNC:28993	ENSG00000103335	Human, mice	Validated	driver
107	MIR15A	HGNC:31543	ENSG00000283785	Mice	Validated	driver
108	EGR1	HGNC:3238	ENSG00000120738	Mice	Validated	driver
109	KDM6B	HGNC:29012	ENSG00000132510	Human, mice	Validated	driver
110	KDM5C	HGNC:11114	ENSG00000126012	Human, mice	Validated	driver
111	MEG3	HGNC:14575	ENSG00000214548	Mice	Validated	driver
112	CCDC6	HGNC:18782	ENSG00000108091	Human, mice	Validated	driver
113	CFL1	HGNC:1874	ENSG00000172757	Mice	Validated	driver
114	ALOXE3	HGNC:13743	ENSG00000179148	Mice	Validated	driver
115	KMT2D	HGNC:7133	ENSG00000167548	Mice	Validated	driver
116	GPX4	HGNC:4556	ENSG00000167468	Human, mice	Validated	suppressor
117	RB1	HGNC:9884	ENSG00000139687	Human, mice	Validated	suppressor
118	HSPB1	HGNC:5246	ENSG00000106211	Human, mice	Validated	suppressor
119	HSF1	HGNC:5224	ENSG00000185122	Human, mice	Validated	suppressor
120	SLC7A11	HGNC:11059	ENSG00000151012	Human, mice	Validated	suppressor
121	GCLC	HGNC:4311	ENSG00000001084	Mice	Validated	suppressor
122	NFE2L2	HGNC:7782	ENSG00000116044	Human, mice	Validated	suppressor
123	SQSTM1	HGNC:11280	ENSG00000161011	Human, mice	Validated	suppressor
124	NQO1	HGNC:2874	ENSG00000181019	Human, mice	Validated	suppressor
125	HMOX1	HGNC:5013	ENSG00000100292	Human, mice	Validated	suppressor
126	FTH1	HGNC:3976	ENSG00000167996	Human, mice	Validated	suppressor
127	SLC3A2	HGNC:11026	ENSG00000168003	Mice	Validated	suppressor
128	MT1G	HGNC:7399	ENSG00000125144	Human, mice	Validated	suppressor
129	FANCD2	HGNC:3585	ENSG00000144554	Mice	Validated	suppressor
130	FTMT	HGNC:17345	ENSG00000181867	Mice, fly	Validated	suppressor
131	HSPA5	HGNC:5238	ENSG00000044574	Human, mice	Validated	suppressor
132	TP53	HGNC:11998	ENSG00000141510	Human, mice	Validated	suppressor

© 2023. Xu J et al. Published online at http://www.aginganddisease.org/EN/10.14336/AD.2023.0509

133	HELLS	HGNC:4861	ENSG00000119969	Human, mice	Validated	suppressor
134	MTOR	HGNC:3942	ENSG00000198793	Mice	Validated	suppressor
135	MIR137	HGNC:31523	ENSG00000284202	Human, mice	Validated	suppressor
136	SLC40A1	HGNC:10909	ENSG00000138449	Mice	Validated	suppressor
137	CBS	HGNC:1550	ENSG00000160200	Human, mice	Validated	suppressor
138	OTUB1	HGNC:23077	ENSG00000167770	Human, mice	Validated	suppressor
139	SCD	HGNC:10571	ENSG00000099194	Human, mice	Validated	suppressor
140	SESN2	HGNC:20746	ENSG00000130766	Human, mice	Validated	suppressor
141	NF2	HGNC:7773	ENSG00000186575	Human, mice	Validated	suppressor
142	ARNTL	HGNC:701	ENSG00000133794	Human, mice	Validated	suppressor
143	HIF1A	HGNC:4910	ENSG00000100644	Human, mice	Validated	suppressor
144	JUN	HGNC:6204	ENSG00000177606	Human, mice	Deduced	suppressor
145	TMBIM4	HGNC:24257	ENSG00000155957	Human, mice	Validated	suppressor
146	PLIN2	HGNC:248	ENSG00000147872	Human, mice	Screened	suppressor
147	MIR212	HGNC:31589	ENSG00000267195	Mice	Validated	suppressor
148	AIFM2	HGNC:21411	ENSG00000042286	Human, mice	Validated	suppressor
149	ZFP36	HGNC:12862	ENSG00000128016	Human, mice	Validated	suppressor
150	CHMP5	HGNC:26942	ENSG00000086065	Human, mice	Validated	suppressor
151	CHMP6	HGNC:25675	ENSG00000176108	Human, mice	Validated	suppressor
152	CAV1	HGNC:1527	ENSG00000105974	Human, mice	Validated	suppressor
153	PIR	HGNC:30048	ENSG00000087842	Human, mice	Validated	suppressor
154	HCAR1	HGNC:4532	ENSG00000196917	Human, mice	Validated	suppressor
155	SLC16A1	HGNC:10922	ENSG00000155380	Human, mice	Validated	suppressor
156	FZD7	HGNC:4045	ENSG00000155760	Human, mice	Validated	suppressor
157	PLA2G6	HGNC:9039	ENSG00000184381	Human, mice	Validated	suppressor
158	PARK7	HGNC:16369	ENSG00000116288	Human, mice	Validated	suppressor
159	STAT3	HGNC:11364	ENSG00000168610	Mice	Validated	suppressor
160	ACOT1	HGNC:33128	ENSG00000184227	Mice	Validated	suppressor
161	ALDH3A2	HGNC:403	ENSG00000072210	Mice	Validated	suppressor
162	FNDC5	HGNC:20240	ENSG00000160097	Human, mice	Deduced	suppressor
163	CDH1	HGNC:1748	ENSG00000039068	Human, mice	Validated	suppressor
164	MIR214	HGNC:31591	ENSG00000283844	Human, mice	Validated	suppressor
165	TF	HGNC:11740	ENSG00000091513	Human, mice	Validated	suppressor
166	GLRX5	HGNC:20134	ENSG00000182512	Human, mice	Validated	suppressor
167	RHEBP1	HGNC:10010	ENSG00000229927	Human, mice	Validated	suppressor
168	PPP1R13L	HGNC:18838	ENSG00000104881	Mice	Validated	suppressor
169	IDH2	HGNC:5383	ENSG00000182054	Human, mice	Validated	suppressor
170	NOS2	HGNC:7873	ENSG00000007171	Mice	Validated	suppressor
171	RELA	HGNC:9955	ENSG00000173039	Mice	Validated	suppressor
172	VDR	HGNC:12679	ENSG00000111424	Mice	Validated	suppressor
173	FXN	HGNC:3951	ENSG00000165060	Mice	Validated	suppressor
174	TXN	HGNC:12435	ENSG00000136810	Mice	Validated	suppressor
175	FGF21	HGNC:3678	ENSG00000105550	Mice	Validated	suppressor
176	TFRC	HGNC:11763	ENSG00000072274	Mice	Validated	suppressor
177	ASAH2	HGNC:18860	ENSG00000188611	Mice	Validated	suppressor

© 2023. Xu J et al. Published online at http://www.aginganddisease.org/EN/10.14336/AD.2023.0509

178	TYRO3	HGNC:12446	ENSG00000092445	Human, mice	Validated	suppressor
179	ECH1	HGNC:3149	ENSG00000104823	Human, mice	Validated	suppressor
180	PRDX6	HGNC:16753	ENSG00000117592	Mice	Validated	suppressor
181	CHMP1A	HGNC:8740	ENSG00000131165	Mice	Validated	suppressor
182	SOX2	HGNC:11195	ENSG00000181449	Human, mice	Validated	suppressor
183	PROK2	HGNC:18455	ENSG00000163421	Mice	Validated	suppressor
184	SIRT2	HGNC:10886	ENSG00000068903	Mice	Validated	suppressor
185	circRNA1615	_NA_	_NA_	Mice	Validated	suppressor
186	SMPD1	HGNC:11120	ENSG00000166311	Mice	Validated	suppressor
187	ADAMTS13	HGNC:1366	ENSG00000160323	Mice	Validated	suppressor
188	CircFNDC3B	_NA_	_NA_	Mice	Validated	suppressor
189	PPARD	HGNC:9235	ENSG00000112033	Mice	Validated	suppressor
190	ENO3	HGNC:3354	ENSG00000108515	Mice	Validated	suppressor
191	MIR9-3HG	HGNC:27388	ENSG00000255571	Mice	Validated	suppressor
192	ADIPOQ	HGNC:13633	ENSG00000181092	Mice	Validated	suppressor
193	mmu_circRNA_ 0000309	_NA_	_NA_	Mice	Validated	suppressor
194	MS4A15	HGNC:28573	ENSG00000166961	Mice	Validated	suppressor
195	FURIN	HGNC:8568	ENSG00000140564	Human, mice	Deduced	suppressor
196	circGFRA1	_NA_	_NA_	Human, mice	Validated	suppressor
197	MAPKAP1	HGNC:18752	ENSG00000119487	Mice	Deduced	suppressor
198	MLST8	HGNC:24825	ENSG00000167965	Mice	Deduced	suppressor
199	PRR5	HGNC:31682	ENSG00000186654	Mice	Deduced	suppressor
200	RICTOR	HGNC:28611	ENSG00000164327	Mice	Deduced	suppressor
201	TERT	HGNC:11730	ENSG00000164362	Mice	Validated	suppressor

© 2023. Xu J et al. Published online at http://www.aginganddisease.org/EN/10.14336/AD.2023.0509

SUPPLEMENTARY DATA

Supplementary Table 2. Antibody information

Primary antibodies				
**Protein**	Application	Catalog Number	Host species	Company
**4-HNE**	IF/IHC 1:200; WB 1:1000	ab48506	mouse	Abcam
**CTSB**	IF/IHC 1:400; WB 1:1000	ab214428	rabbit	Abcam
**GFAP**	IF 1:400	16825-1-AP	rabbit	Proteintech
**GFAP**	IF 1:200	60190-1-lg	mouse	Proteintech
**F4/80**	IF 1:200	ab6640	rat	Abcam
**IBA1**	IF 1:150	GTX89792	goat	Genetex
**CD11b**	IF 1:400	66519-1-lg	mouse	Proteintech
**CD11b**	IF 1:400	FITC-65055	rat	Proteintech
**LAMP1**	IF 1:100	sc-20011	mouse	Santa cruz
**mtTFA**	WB 1:500	sc-166965	mouse	Santa cruz
**ACSL4**	WB 1:10000	ab155282	rabbit	Abcam
**β-actin**	WB 1:20000	66009-1-lg	mouse	Proteintech
**CD86**	IF 1:200	91882	rabbit	Cell signaling technology
**CD206**	IF 1:200; WB 1:1000	18704-1-AP	rabbit	Proteintech
**iNOS**	IF 1:200; WB 1:2000	18985-1-AP	rabbit	Proteintech
**Arginase-1**	IF 1:200; WB 1:8000	16001-1-AP	rabbit	Proteintech
**IL-1β**	WB 1:500	511369	rabbit	Zenbio
**Fibronectin1**	IF 1:200	66042-1-lg	mouse	Proteintech
**β3-Tubulin**	IF 1:400	5568	rabbit	Cell signaling technology
**Secondary antibodies**				
**Donkey Anti-Rabbit IgG H&L (Alexa Fluor® 488)**	IF 1:400	ab150073	donkey	Abcam
**Goat Anti-Rat IgG H&L (Alexa Fluor® 488)**	IF 1:400	ab150157	goat	Abcam
**Goat Anti-Mouse IgG H&L (Alexa Fluor® 594)**	IF 1:400	ab150116	goat	Abcam
**Goat Anti-Rabbit IgG H&L (Alexa Fluor® 594)**	IF 1:400	ab150080	goat	Abcam
**Goat Anti-Rat IgG H&L (Alexa Fluor® 594)**	IF 1:400	ab150160	goat	Abcam
**Donkey Anti-Goat IgG H&L (Alexa Fluor® 594)**	IF 1:400	ab150132	donkey	Abcam
**Donkey Anti-Rabbit IgG H&L (Alexa Fluor® 647)**	IF 1:400	ab150075	donkey	Abcam
**Goat Anti-Rabbit IgG(H+L)(peroxidase/HRP conjugated)**	WB 1:10000	E-AB-1003	goat	Elabscience
**Goat Anti-Mouse IgG(H+L)(peroxidase/HRP conjugated)**	WB 1:10000	E-AB-1008	goat	Elabscience

© 2023. Xu J et al. Published online at http://www.aginganddisease.org/EN/10.14336/AD.2023.0509

SUPPLEMENTARY DATA

Supplementary Table 3. Summary of statistical methods

Data sources	Group number	Number of biological replicates (n)	Normality test/ variance test methods	Normality test results	statistical test methods	statistical test results
**Fig .1C**	5	3	Shapiro-Wilk test/ Brown-Forsythe test	Passed (alpha=0.05)/ P=0.32	Kruskal-Wallis test/ Dunn's test	P<0.0001
**Fig .1E**	2	4	Shapiro-Wilk test/F test	Passed (alpha=0.05)/P=0.6668	Unpaired t test	P=0.0271
**Fig .1F**	2	5	Kolmogorov-Smirnov test/F test	Passed (alpha=0.05)/P=0.5514	Unpaired t test	P=0.0010
**Fig .1G**	2	5	Kolmogorov-Smirnov test/F test	Passed (alpha=0.05)/P=0.3759	Unpaired t test	P=<0.0001
**Fig .1H**	2	5	Kolmogorov-Smirnov test/F test	Passed (alpha=0.05)/P=0.2578	Unpaired t test	P=0.0016
**Fig .1J**	5	3	Shapiro-Wilk test/ Brown-Forsythe test	Passed (alpha=0.05)/ P=0.2769	Kruskal-Wallis test/ Dunn's test	P<0.0001
**Fig. 3K**	4	-	Kolmogorov-Smirnov test/ Brown-Forsythe test	Passed (alpha=0.05)/P=0.1450	Ordinary one-way ANOVA/Tukey's multiple comparisons test	F=6.825<br>P=0.0006<br>R squared=0.2947
**Fig .4C**	2	4	Shapiro-Wilk test/-	Not passed/-	Mann-Whitney test	P=0.3419
**Fig .4D**	4	3	Shapiro-Wilk test/ Brown-Forsythe test	Passed (alpha=0.05)/ P=0.6319	Kruskal-Wallis test/ Dunn's test	P=0.0004
**Fig .4F**	5	3	Shapiro-Wilk test/ Brown-Forsythe test	Passed (alpha=0.05)/ P=0.2769	Kruskal-Wallis test/ Dunn's test	P<0.0001
**Fig .5B**	2	3	Shapiro-Wilk test/F test	Passed (alpha=0.05)/P=0.3565	Unpaired t test	P=0.0220
**Fig .5E**	4	4	Shapiro-Wilk test/ Brown-Forsythe test	Passed (alpha=0.05)/ P=0.8433/0.5956/0.2080	Ordinary one-way ANOVA/Tukey's multiple comparisons test	F=35.15/6.449/32.24<br>P<0.0001/0.0076/<0.0001<br>R squared=0.8978/ 0.6172/0.8896
**Fig .5G**	4	4	Shapiro-Wilk test/ Brown-Forsythe test	Passed (alpha=0.05)/ P=0.9182	Ordinary one-way ANOVA/Tukey's multiple comparisons test	F=94.33<br>P<0.0001<br>R squared=0.9593
**Fig .5I**	4	4	Shapiro-Wilk test/ Brown-Forsythe test	Passed (alpha=0.05)/ P=0.2689	Ordinary one-way ANOVA/Tukey's multiple comparisons test	F=30.80<br>P<0.0001<br>R squared=0.8851
**Fig .5K**	4	3	Shapiro-Wilk test/ Brown-Forsythe test	Passed (alpha=0.05)/ P=0.3842	Kruskal-Wallis test/ Dunn's test	P=0.0001
**Fig .6C**	6	3	Shapiro-Wilk test/ Brown-Forsythe test	Passed (alpha=0.05)/ P=0.6082	Kruskal-Wallis test/ Dunn's test	P=0.0054
**Fig .6E**	6	4	Shapiro-Wilk test/ Brown-Forsythe test	Passed (alpha=0.05)/ P=0.1017	Ordinary one-way ANOVA/Tukey's multiple comparisons test	F=10.65<br>P<0.0001<br>R squared=0.7473
**Fig .6G**	5	4	Shapiro-Wilk test/ Brown-Forsythe test	Passed (alpha=0.05)/ P=0.1266/0.5025	Ordinary one-way ANOVA/Tukey's multiple comparisons test	F=114.3/21.21<br>P<0.0001/<0.0001<br>R squared=0.9794/0.8498
**Fig .6I**	5	4	Shapiro-Wilk test/ Brown-Forsythe test	Passed (alpha=0.05)/ P=0.5311/0.2773/0.4901	Ordinary one-way ANOVA/ Tukey's multiple comparisons test	F=21.77/12.67/54.61<br>P<0.0001/=0.0001/<0.0001 R squared=0.8531 /0.7716/0.9357
**Fig .7B**	3	6	Kolmogorov-Smirnov test/ Brown-Forsythe test	Passed (alpha=0.05)/P=0.7154	Ordinary one-way ANOVA/Tukey's multiple comparisons test	F=58.58<br>P<0.0001<br>R squared=0.8865
**Fig .7C**	3	6	Kolmogorov-Smirnov test/ Brown-Forsythe test	Passed (alpha=0.05)/P=0.1182	Ordinary one-way ANOVA/Tukey's multiple comparisons test	F=88.68<br>P<0.0001<br>R squared=0.9220
**Fig .7D**	3	6	Kolmogorov-Smirnov test/ Brown-Forsythe test	Passed (alpha=0.05)/P=0.4650	Ordinary one-way ANOVA/Tukey's	F=53.41<br>P<0.0001<br>R squared=0.8769

© 2023. Xu J et al. Published online at http://www.aginganddisease.org/EN/10.14336/AD.2023.0509

					multiple comparisons test	
**Fig .7F**	2	4	Shapiro-Wilk test/F test	Passed (alpha=0.05)/P=0.2896	Unpaired t test	P=0.0004
**Fig .7H**	3	4	Shapiro-Wilk test/ Brown-Forsythe test	Passed (alpha=0.05)/ P=0.2451/0.1843/0.0628	Ordinary one-way ANOVA/Tukey's multiple comparisons test	F=25.94/21.16/30.85 P=0.0002/0.0004/<0.0001 R squared=0.8521/ 0.8246/0.8727
**Fig .7J**	3	4	Shapiro-Wilk test/ Brown-Forsythe test	Passed (alpha=0.05)/ P=0.4503	Ordinary one-way ANOVA/Tukey's multiple comparisons test	F=6.795 P=0.0159 R squared=0.6016
**Fig. 8B**	2	4	Shapiro-Wilk test/F test	Passed/not passed P=0.2661/-	Unpaired t test/ Mann-Whitney test	P=0.0004 P=0.0286
**Fig. 8D**	3	4	Shapiro-Wilk test/ Brown-Forsythe test	Passed (alpha=0.05)/ P=0.3136/0.0827/0.1335	Ordinary one-way ANOVA/Tukey's multiple comparisons test	F=13.36/40.63/53.33 P=0.0020/<0.0001/<0.0001 R squared=0.7480/ 0.9003/0.9222
**Fig. 8F**	2	3	Shapiro-Wilk test/F test	Passed (alpha=0.05)/ P=0.3918	Unpaired t test	P=0.0193
**Fig. 9A**	3	6	Kolmogorov-Smirnov test	-	Two-way ANOVA/ Tukey's multiple comparisons test	Interaction F=70.48, P<0.0001 Row Factor F=275.5, P<0.0001 Column Factor F=3220, P<0.0001
**Fig. 9B**	3	6	Kolmogorov-Smirnov test	-	Two-way ANOVA/ Tukey's multiple comparisons test	Interaction F=85.66, P<0.0001 Row Factor F=325.3, P<0.0001 Column Factor F=5735, P<0.0001
**Fig. 9C**	3	6	Kolmogorov-Smirnov test	-	Two-way ANOVA/ Tukey's multiple comparisons test	Interaction F=15.91, P<0.0001 Row Factor F=1364, P<0.0001 Column Factor F=83.16, P<0.0001
**Fig. 9E**	3	6	Kolmogorov-Smirnov test/ Brown-Forsythe test	Passed (alpha=0.05)/ P=0.0548	Ordinary one-way ANOVA/ Tukey's multiple comparisons test	F=1110.9 P<0.0001 R squared=0.9367
**Fig. 9G**	3	6	Kolmogorov-Smirnov test/Brown-Forsythe test	Passed (alpha=0.05)/ P=0.6883	Ordinary one-way ANOVA/Tukey's multiple comparisons test	F=160.8 P<0.0001 R squared=0.9554
**Fig. 9J**	2	6	Kolmogorov-Smirnov test	Not passed	Mann-Whitney test	P=0.0022
**Fig. 9K**	2	6	Kolmogorov-Smirnov test/F test	Passed (alpha=0.05)/ P=0.5144	Unpaired t test	P=0.0020
**Fig .S1D**	2	5	Kolmogorov-Smirnov test/F test	Passed (alpha=0.05)/P<0.0001	Welch's test	P<0.0001
**Fig .S4B**	5	3	Shapiro-Wilk test/ Brown-Forsythe test	Passed (alpha=0.05)/ P=0.8101	Kruskal-Wallis test/ Dunn's test	P<0.0001
**Fig .S5A**	5	5	Kolmogorov-Smirnov test /Brown-Forsythe test	Passed (alpha=0.05)/ P=0.1339	Ordinary one-way ANOVA	F=11.75 P<0.0001 R squared=0.7015
**Fig .S5D**	5	5	Kolmogorov-Smirnov test /Brown-Forsythe test	Passed (alpha=0.05)/ P=0.2017	Ordinary one-way ANOVA	F=383.4 P<0.0001 R squared=0.9871
**Fig .S5E**	5	6	Kolmogorov-Smirnov test /Brown-Forsythe test	Passed (alpha=0.05)/ P=0.7460	Ordinary one-way ANOVA/Tukey's multiple comparisons test	F=37.43 P<0.0001 R squared=0.8569
**Fig .S7C**	3	3	Shapiro-Wilk test/ Brown-Forsythe test	Passed (alpha=0.05)/ P=0.2552/0.2076/0.5038	Kruskal-Wallis test/ Dunn's test	P=0.0714 P=0.0714 P=0.0036
**Fig. S8B**	2	4	Shapiro-Wilk test/F test	Passed/passed P=0.5354/0.0257	Unpaired t test/Welch's test	P=0.0348 P=0.0160

© 2023. Xu J et al. Published online at http://www.aginganddisease.org/EN/10.14336/AD.2023.0509

SUPPLEMENTARY DATA

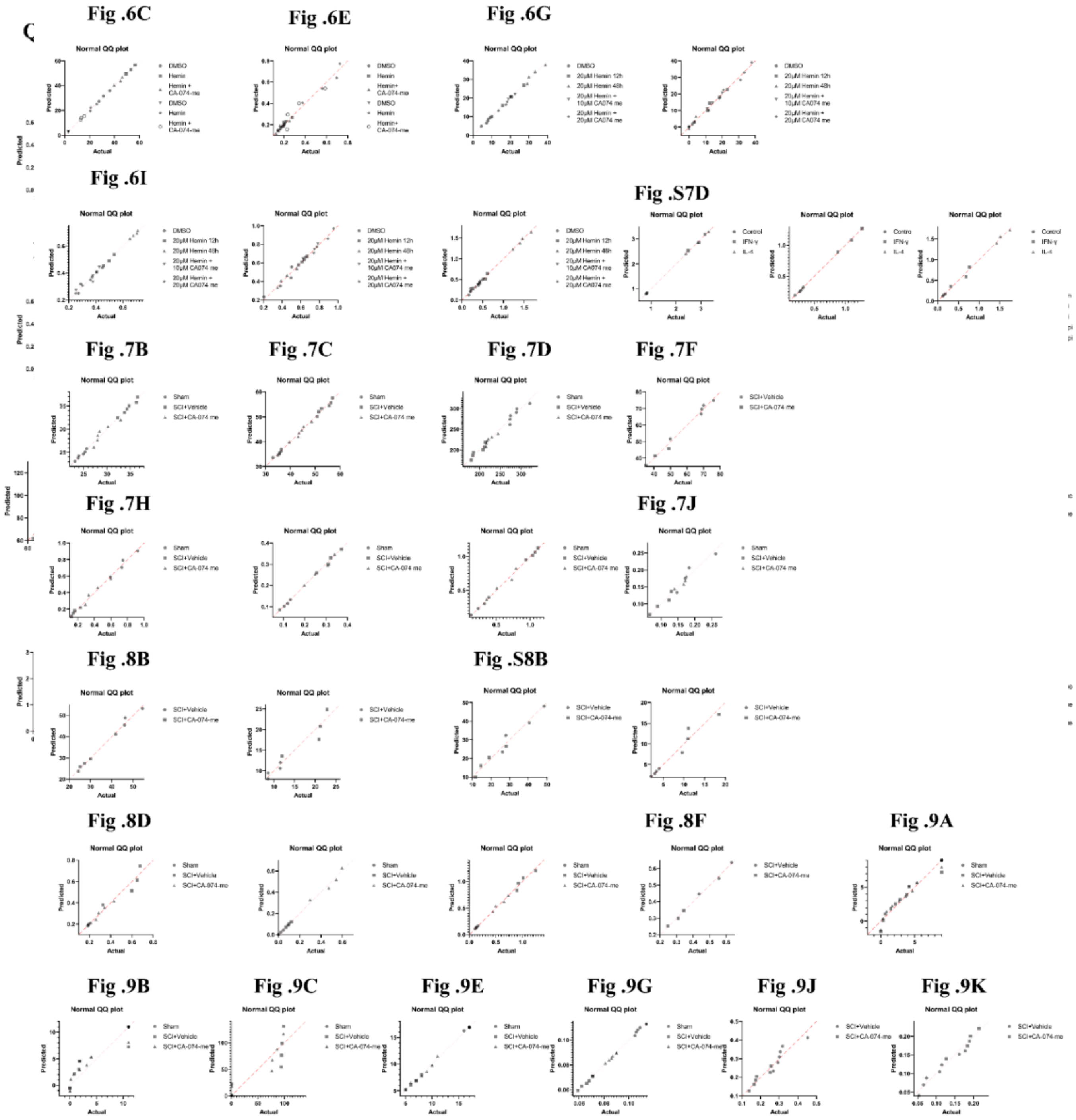

© 2023. Xu J et al. Published online at http://www.aginganddisease.org/EN/10.14336/AD.2023.0509

SUPPLEMENTARY DATA

Supplementary Table 4. Differentially expressed ferroptosis driver/suppressor genes from five bulk RNA sequencing transcriptome profiles

	180767 4h	5296 0.5h	5296 4h	5296 28d	5296 47681 7d	5296 47681 24h	5296 47681 72h	45376 2d	45376 7d	46695 3w
DRIVER										
1	Il6	Slc38a1	Slc38a1	Scp2	Slc1a5	Flt3	Slc38a1	Flt3	Flt3	Flt3
2		Slc1a5	Slc1a5	Slc38a1	Atg5	Atg5	Slc1a5	Slc38a1	Slc1a5	Il1b
3		Atg5	Atg5	Slc1a5	Ulk1	Snx4	Atg5	Gls2	Gls2	Trim21
4		Ulk1	Gabarapl1	Atg5	Snx4	Alox15	Ulk1	Alox5	Got1	
5		Gabarapl1	Bid	Ulk1	Ulk2	Bid	Gabarapl1	G6pdx	Alox5	
6		Ulk2	Pebp1	Gabarapl1	Sat1	Dpp4	Atg13	Ulk1	G6pdx	
7		Bid	Mapk14	Snx4	Pebp1	Pebp1	Ulk2	Map1lc3a	Ulk1	
8		Dpp4	Lpin1	Ulk2	Hilpda	Mapk14	Sat1	Sat1	Atg4d	
9		Pebp1	Egln2	Sat1	Egln2	Panx1	Pebp1	Lpcat3	Gabarapl1	
10		Mapk14	Miox	Bid	Panx1	Por	Mapk14	Bid	Atg16l1	
11		Egln2	Panx1	Pebp1	Por	Ctsb	Lpin1	Mapk14	Wipi2	
12		Tafazzin	Por	Mapk14	Nr1d2	Trim21	Egln2	Ano6	Snx4	
13		Panx1	Nr1d2	Egln2	Trim21	Gja1	Miox	Lpin1	Atg13	
14		Por	Ctsb	Miox	Gja1	Usp11	Tafazzin	Fbxw7	Sat1	
15		Nr1d2	Nox4	Tafazzin			Panx1	Panx1	Lpcat3	
16		Ctsb	Trim21	Sirt1			Nr1d2	Por	Bid	
17		Gja1	Gja1	Panx1			Zeb1	Nr1d1	Cdkn2a	
18		Egr1		Por			Tsc1	Nr1d2	Lpin1	
19				Nr1d2			Pparg	Ctsb	Fbxw7	
20				Ctsb			Nox4	Atf4	Nr1d1	
21				Zeb1			Dpep1	Pvt1	Nr1d2	
22				Tsc1			Kdm5c	Slc39a14	Ctsb	
23				Ifna4				Tsc1	Map3k11	
24				Pparg				Lgmn	Brd7	
25				Trim21				Map3k14	Tsc1	
26				Gja1				Trim21	Lgmn	
27				Kdm5c				Gja1	Map3k14	
28								Usp11	Mib2	
29								Egr1	Trim21	
30								Kdm5c	Usp11	
31								Meg3	Meg3	
SUPPRESSOR										
1	Hspb1	Rb1	Nfe2l2	Slc7a11	Hspa5	Hspb1	Slc7a11	Hspb1	Nfe2l2	
2		Nfe2l2	Hspa5	Nfe2l2	Otub1	Nfe2l2	Hells	Gclc	Slc3a2	
3		Hspa5	Hells	Ftmt	Jun	Hells	Slc40a1	Nfe2l2	Mtor	
4		Mtor	Mtor	Hspa5	Slc16a1	Jun	Otub1	Nqo1	Slc40a1	
5		Slc40a1	Slc40a1	Mtor	Aldh3a2	Stat3	Jun	Hspa5	Cbs	
6		Otub1	Otub1	Slc40a1	Rela	Aldh3a2	Park7	Cbs	Otub1	
7		Slc16a1	Jun	Jun	Sox2	Vdr	Aldh3a2	Nf2	Jun	
8		Park7	Slc16a1	Slc16a1	Furin	Fxn	Nos2	Jun	Plin2	
9		Aldh3a2	Aldh3a2	Park7	Mlst8	Sox2	Vdr	Plin2	Aifm2	

© 2023. Xu J et al. Published online at http://www.aginganddisease.org/EN/10.14336/AD.2023.0509

10	Sox2	Rela	Aldh3a2		Eno3	Sox2	Zfp36	Zfp36
11	Eno3	Vdr	Vdr			Prok2	Chmp6	Chmp5
12	Mapkap1	Sox2	Prok2			Furin	Slc16a1	Fzd7
13	Mlst8	Eno3	Mapkap1			Mlst8	Fzd7	Stat3
14		Mapkap1	Mlst8				Stat3	Fndc5
15		Mlst8					Aldh3a2	Idh2
16							Fndc5	Rela
17							Ppp1r13l	Asah2
18							Idh2	Tyro3
19							Rela	Ech1
20							Asah2	Prdx6
21							Sox2	Chmp1a
22							Ppard	Ppard
23							Furin	Furin
24							Prr5	Mlst8
BOTH								
1		Hif1a	Hif1a	Hif1a			Hmox1	Tfrc
2								Hmox1

© 2023. Xu J et al. Published online at http://www.aginganddisease.org/EN/10.14336/AD.2023.0509

SUPPLEMENTARY DATA

Supplementary Table 5. Differentially expressed ferroptosis driver/suppressor genes in acute, subacute, and chronic phases

	DRIVER					
	only acute	only subacute	only chronic	acute| subacute	acute| chronic	acute| subacute| chronic
1	Il6	Hilpda	Scp2	Lpin1	Slc38a1	Slc1a5
2	Dpp4	Got1	Sirt1	Usp11	Mapk14	Atg5
3	Egr1	Atg4d	Ifna4	Atg13	Tafazzin	Ulk1
4	Nox4	Atg16l1	Il1b	Gls2	Miox	Gabarapl1
5	Alox15	Wipi2		Alox5	Zeb1	Ulk2
6	Dpep1	Cdkn2a		G6pdx	Pparg	Bid
7	Map1lc3a	Map3k11		Lpcat3	Kdm5c	Pebp1
8	Ano6	Brd7		Fbxw7		Egln2
9	Atf4	Mib2		Nr1d1		Panx1
10	Pvt1	Tfrc		Lgmn		Por
11	Slc39a14			Map3k14		Nr1d2
12				Meg3		Ctsb
13				Hmox1		Gja1
14						Trim21
15						Hif1a
16						Flt3
17						Snx4
18						Sat1
19						Tsc1

	SUPPRESSOR					
	only acute	only subacute	only chronic	acute| subacute	acute| chronic	acute| subacute| chronic
1	Hspb1	Slc3a2	Ftmt	Otub1	Park7	Nfe2l2
2	Rb1	Aifm2		Sox2	Mapkap1	Hspa5
3	Eno3	Chmp5		Rela	Vdr	Mtor
4	Hells	Tyro3		Stat3	Hif1a	Slc40a1
5	Fxn	Ech1		Furin	Slc7a11	Slc16a1
6	Nos2	Prdx6		Cbs	Prok2	Aldh3a2
7	Gclc	Chmp1a		Plin2		Mlst8
8	Nqo1	Tfrc		Zfp36		Jun
9	Nf2			Fzd7		
10	Chmp6			Fndc5		
11	Ppp1r13l			Idh2		
12	Prr5			Asah2		
13				Ppard		
14				Hmox1		

© 2023. Xu J et al. Published online at http://www.aginganddisease.org/EN/10.14336/AD.2023.0509
